# Binding site plasticity in viral PPxY Late domain recognition by the third WW domain of human NEDD4

**DOI:** 10.1038/s41598-019-50701-3

**Published:** 2019-10-21

**Authors:** Manuel Iglesias-Bexiga, Andrés Palencia, Carles Corbi-Verge, Pau Martin-Malpartida, Francisco J. Blanco, Maria J. Macias, Eva S. Cobos, Irene Luque

**Affiliations:** 1 Department of Physical Chemistry and Institute of Biotechnology, University of Granada, 18071 Granada, Spain; 2 Donnelly Centre for Cellular and Biomolecular Research, Faculty of Medicine, University of Toronto, Toronto, Ontario Canada; 3 Institute for Research in Biomedicine (IRB Barcelona), The Barcelona Institute of Science and Technology (BIST). Baldiri Reixac, 10, Barcelona, 08028 Spain; 4 Structural Biology Unit, CIC bioGUNE, Parque Tecnológico de Bizkaia, 48160 Derio, Spain; 5 IKERBASQUE, Basque Foundation for Science, María Díaz de Haro 3, 48013 Bilbao, Spain; 6 ICREA, Passeig Lluís Companys 23, 08010 Barcelona, Spain; 7 Present Address: Instituto de Química-Física Rocasolano, Consejo Superior de Investigaciones Científicas, C/Serrano 119, 28006 Madrid, Spain; 8 Present Address: Institute for Advanced Biosciences, Structural Biology of Novel Targets in Human Diseases Group, Inserm U1209–CNRS 5309–Université Grenoble-Alpes, 38700 La Tronche, France

**Keywords:** Molecular conformation, Thermodynamics, Intracellular signalling peptides and proteins, Viral proteins, NMR spectroscopy

## Abstract

The recognition of PPxY viral Late domains by the third WW domain of the HECT-E3 ubiquitin ligase NEDD4 (hNEDD4-WW3) is essential for the completion of the budding process of numerous enveloped viruses, including Ebola, Marburg, HTLV1 or Rabies. hNEDD4-WW3 has been validated as a promising target for the development of novel host-oriented broad spectrum antivirals. Nonetheless, finding inhibitors with good properties as therapeutic agents remains a challenge since the key determinants of binding affinity and specificity are still poorly understood. We present here a detailed structural and thermodynamic study of the interactions of hNEDD4-WW3 with viral Late domains combining isothermal titration calorimetry, NMR structural determination and molecular dynamics simulations. Structural and energetic differences in Late domain recognition reveal a highly plastic hNEDD4-WW3 binding site that can accommodate PPxY-containing ligands with varying orientations. These orientations are mostly determined by specific conformations adopted by residues I859 and T866. Our results suggest a conformational selection mechanism, extensive to other WW domains, and highlight the functional relevance of hNEDD4-WW3 domain conformational flexibility at the binding interface, which emerges as a key element to consider in the search for potent and selective inhibitors of therapeutic interest.

## Introduction

Human NEDD4 (hNEDD4) is a HECT type E3 ubiquitin ligase widely expressed and evolutionarily conserved in eukaryotes. hNEDD4 is one of the nine members of the NEDD family of E3 ubiquitin ligases, characterized by a conserved modular architecture consisting of an N-Terminal C2 Ca^2+^-regulated lipid binding domain, 2 to 4 WW protein interaction domains in charge of substrate recognition through the interaction with PY containing sequences, and a highly conserved HECT catalytic domain^1,2^. hNEDD4 functions within the ubiquitin proteasome system regulating trafficking and stability of signaling proteins implicated in many cellular processes, including sodium homeostasis, T-cell regulation, control of neuronal function and cellular growth and proliferation^1,2^. Its abnormal activity has been associated to the development of cancer and hypertension^3,4^. hNEDD4 is also implicated in the progression of viral infection, playing an essential role in the budding process of encapsulated viruses, including filoviruses (Ebola, Marburg), rhabdoviruses (Lassa) or arenaviruses (Rabies)^5,6^, through its interactions with viral Late domains.

Viral L- or Late domains are short and conserved sequences found in the *gag* polyproteins of retroviruses and in the matrix proteins of filoviruses (VP40), arenaviruses (Z) and rhabdoviruses (M) in different numbers and combinations. Three types of Late domains have been described, characterized by different core motifs (PPxY, P(T/S)AP and LYP(x)_n_L), that are interchangeable between divergent viruses and active in a context-independent manner^7^. Viral Late domains function by recruiting the cellular membrane scission machinery (i.e. the ESCRT pathway) to the viral site of budding at the plasma membrane through the establishment of direct interactions with several host factors: P(T/S)AP Late domains interact with the UEV domain of Tsg101, LYP(x)_n_L Late domains with the V domain of the adaptor protein Alix and PPxY Late domains bind to the third WW domain of hNEDD4^5,8,9^. Mutation or deletion of viral Late domains results in the accumulation of immature viral particles that remain attached to the cell, halting the infection^6,7^, and small molecule inhibitors targeting Late domain interactions with their cellular targets can efficiently block egress of a number of viruses^10,11^. Thus, hNEDD4-WW3 constitutes an attractive target for the development of novel, host-oriented, broad-spectrum antivirals with low susceptibility to the development of drug resistance.

WW domains are small protein modules (30–40 amino acids) that fold into a stable three-stranded antiparallel β-sheet^12,13^. WW domains are named after two conserved tryptophan residues spaced 20 to 22 residues apart within the sequence: the first tryptophan lies on one side of the β-sheet conforming a hydrophobic core important for domain stability; the second tryptophan is located at one hydrophobic pocket at the binding site, the xP pocket, dedicated to poly-proline recognition and common to other proline interaction modules (SH3 and UEV domains). WW domains have been classified according to the motifs they recognize. A second pocket in the binding site is responsible for binding specificity within WW domains subfamilies, and, in the case of type-1 WW domains, such as hNEDD4-WW3, it is dedicated to the recognition of the Tyr residue in the PPxY consensus core-motif^14,15^. The search for high affinity and specificity ligands of WW domains with good properties as therapeutic agents and limited secondary effects is challenging due to the promiscuity and low affinity of their natural interactions, which, as in other polyproline recognition modules such as SH3 or UEV domains, involve shallow and relatively featureless interfaces. Thus, developing efficient inhibitors of hNEDD4-WW3/Late domain interactions with good potential as novel antivirals requires a profound understanding of the rules governing molecular recognition in these systems^16–19^, which, in spite of the wealth of structural and functional studies on WW domains, remains to be elucidated.

Here we present a detailed structural and thermodynamic study of the interactions between hNEDD4-WW3 and a set of viral Late domains combining isothermal titration calorimetry, NMR structural determination and molecular dynamics simulations. We show that short Late domain sequences from different viruses bind with low to moderate binding affinity and good selectivity to hNEDD4-WW3, although large differences (up to 20 kJ·mol^−1^) in the enthalpic and entropic contributions to the binding Gibbs energy are found for the different ligands, reflecting heterogeneity in the underlying balance of forces driving recognition. The NMR structures of the Ebola and HTLV1 complexes, representative of the two extreme thermodynamic behaviors, confirm this heterogeneity. They also reveal conformational differences at the binding site that modulate the size and shape of the xP and xY pockets, inducing changes in the interaction patterns and different orientations of the two ligands on the hNEDD4-WW3 surface. Molecular dynamics simulations confirm a high conformational variability at the binding site of apo-NEDD4-WW3 that is drastically reduced upon binding of the different ligands, each stabilizing different binding site geometries. This suggests a conformational selection mechanism, further sustained by the analysis of the structural database of WW complexes, highlighting the high plasticity of the hNEDD4-WW3 binding site, which is determined mostly by residues I859 and T866. Together, our results highlight the functional relevance of the conformational plasticity of hNEDD4-WW3 that emerges as a key element to consider in the search for potent and selective inhibitors of therapeutic interest.

## Results

### Thermodynamic analysis of the binding of Late domain peptides to hNEDD4-WW3

**Figure 1:**
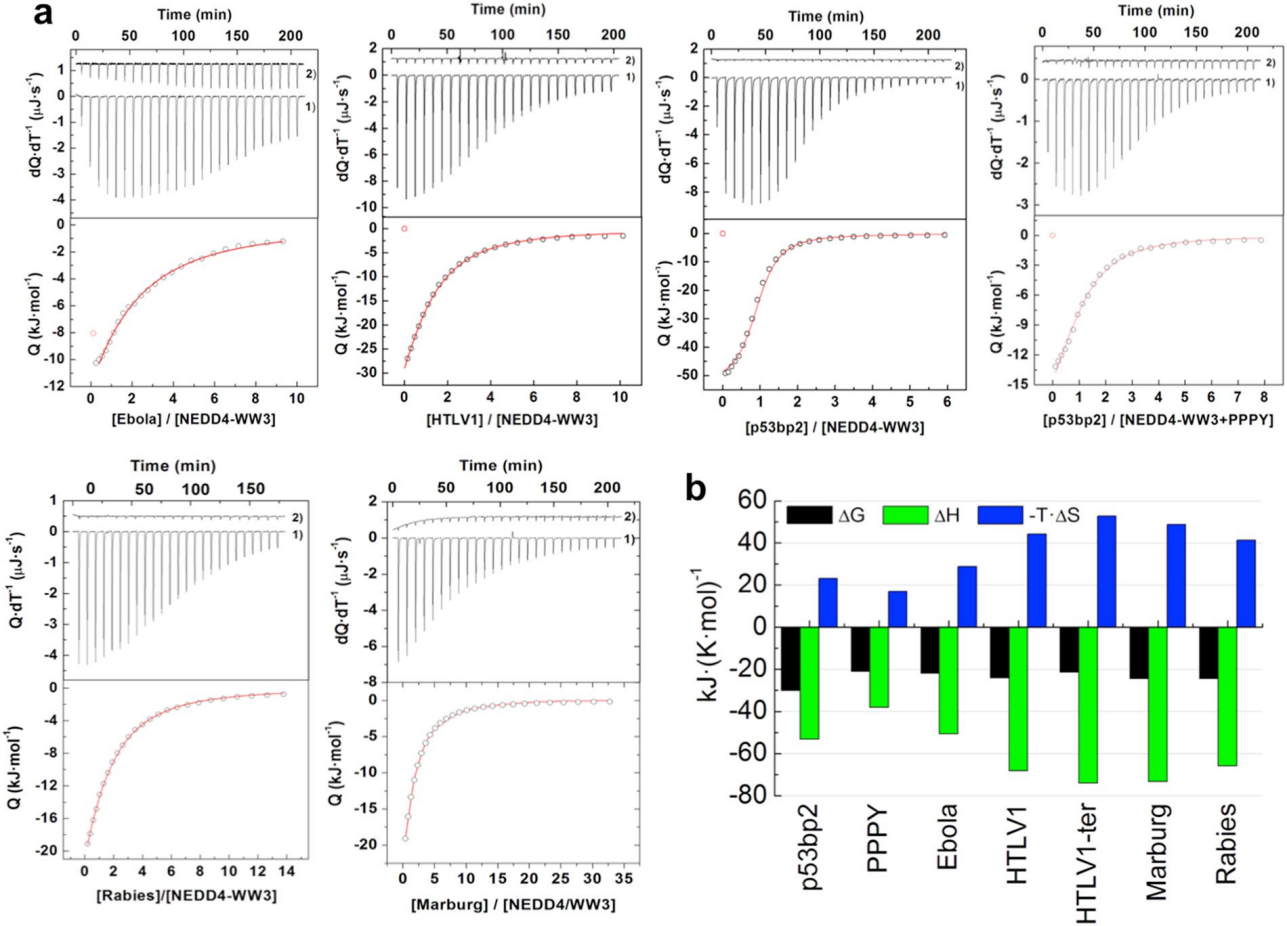
Binding energetics of Late domain peptides to hNEDD4-WW3. (**a**) Calorimetric titrations of peptide ligands into the hNEDD4-WW3 domain. Upper panels show the heat effects associated with the injection of each ligand into the calorimetric cell containing the hNEDD4-WW3 domain (1) and the dilution experiment of each ligand into the corresponding buffer under the same conditions and identical injection profile (2). The dilution curve has been displaced in the y-axis for representation purposes. Lower panels show the binding isotherm (open symbols) together with the best fit (continuous lines) to an appropriate binding model (see Methods for details). (**b**) Thermodynamic dissection of the binding energetics. Shown is the binding Gibbs energy (black bars) as well as the enthalpic (green bars) and entropic (blue bars) contributions. All parameters were determined at 25 °C in 20 mM sodium phosphate pH 7.0.

The binding energetics of hNEDD4-WW3 to a set of five peptide ligands corresponding to the Late domain sequences of different viruses (Supplementary Table S1) were directly measured by Isothermal Titration Calorimetry (ITC) (Fig. 1). The p53bp2 human peptide, previously described to interact with moderate to high binding affinity with several WW domains^20,21^, and the isolated PPPY motif were included in the analysis as a reference. To probe the level of selectivity encoded within these Late domain sequences, binding of the different ligands to the class I WW1 and WW2 domains from YAP was also studied. The results of the thermodynamic analysis are summarized in Table 1.

**Table 1:** Binding energetics of peptide ligands to the hNEDD4-WW3, hYAP-WW1 and hYAP-WW2 domains.

Ligand/sequence	WW domain	T (°C)	^&^K_d_ (μM)	^&^ΔH_ap_ (kJ mol^−1^)	ΔC_p_ (kJ K^−1^ mol^−1^)
**p53bp2** EYPPYP**P**_**−3′**_**P**_**−2′**_**PY**_**0**′_PSG	**NEDD4-WW3**	20	4.1 ± 0.2	−45.6 ± 0.3	−1.59 ± 0.01
		25	5.3 ± 0.5	−53.2 ± 0.7	
		30	9.1 ± 0.3	−61.1 ± 0.6	
	**YAP-WW1**	20	1.1 ± 0.5	−54.8 ± 0.2	−1,16 ± 0.06
		25	1.8 ± 0.1	−61.1 ± 0.3	
		30	3.0 ± 0.1	−66.4 ± 0.5	
	**YAP-WW2**	20	—	—	—
		25	12.0 ± 0.6	−57 ± 1	
		30	—	—	
**HTLV1** SDPQI**P**_**−3′**_**P**_**−2′**_**PY**_**0**′_VEP	**NEDD4-WW3**	25	61 ± 1	−68.2 ± 0.6	—
	**YAP-WW1**	25	^#^308 ± 4	—	
	**YAP-WW2**	25	^#^260 ± 40	—	
**HTLV1**_**ter**_ **P**_**−3′**_**P**_**−2′**_**PY**_**0**′_VEPTAP	**NEDD4-WW3**	25	178 ± 3	−74.0 ± 0.8	—
	**YAP-WW1**	25	n. b.	—	
	**YAP-WW2**	25	n. b.	—	
**Ebola** ILPTA**P**_**−3′**_**P**_**−2′**_**EY**_**0′**_MEA	**NEDD4-WW3**	25	147 ± 4	−50.7 ± 0.7	—
	**YAP-WW1**	25	^#^750 ± 10	—	
	**YAP-WW2**	25	^#^560 ± 30	—	
**Ebola**_**ter**_ ILPTA**P**_**−3**_**P**_**−2′**_**EY**_**0′**_	**NEDD4-WW3**	25	n. b.	—	—
	**YAP-WW1**	25	n. b.	—	
	**YAP-WW2**	25	n. b.	—	
**Marburg** MQYLN**P**_**−3′**_**P**_**−2′**_**PY**_**0′**_ADH	**NEDD4-WW3**	25	51 ± 1	−73.2 ± 0.7	—
	**YAP-WW1**	25	^#^67 ± 4	—	
	**YAP-WW2**	25	^#^17 ± 1	—	
**Rabies** DLWLP**P**_**−3′**_**P**_**−2′**_**EY**_**0′**_VPL	**NEDD4-WW3**	25	51 ± 1	−65.8 ± 0.8	—
	**YAP-WW1**	25	181 ± 5	−63.7 ± 0.9	
	**YAP-WW2**	25	n. b.	—	
**PPPY** **P**_**−3′**_ **P**_**−2′**_ **PY**_**0′**_	**NEDD4-WW3**	25	210 ± 15	^*^−38 ± 1	—
	**YAP-WW1**	25	320 ± 30	^*^−39 ± 3	
	**YAP-WW2**	25	n. b.	—	

^#^Dissociation constants determined by titration experiments followed by fluorescence spectroscopy.

*Thermodynamic parameters obtained by ITC competitive experiments using the p53bp2 ligand.

n. b.: no binding.

Late domain peptides selected to contain the PPxY motif centered in the sequence bind to hNEDD4-WW3 with dissociation constants close to 50 μM, with the exception of the Ebola ligand that shows a lower binding affinity (K_d_ = 147 μM). Placing the PPxY motif at the C-terminal end of the ligands leads to a marked reduction in binding affinity: the dissociation constant for the Human T-Cell Leukaemia Virus-1 Late domain (HTLV1_ter_) drops to values similar to those measured for the isolated PPPY motif (K_d_ = 201 μM) and no binding could be detected for the Ebola_ter_ ligand, neither by ITC nor by fluorescence spectroscopy. In terms of binding selectivity, we find that, while the cellular peptide p53bp2 and the isolated PPPY motif do not distinguish between the three WW domains, all Late domain peptides bind preferentially to their cellular target, hNEDD4-WW3. Moreover, the interaction of these ligands with the YAP WW domains was, in some instances, non detectable and, at best, similar to the non-specific interaction of the isolated PPPY motif, with dissociation constants ranging between 200 and 800 μM. Together, these results confirm that the isolated Late domain sequences maintain the ability to interact with hNEDD4-WW3 and suggest that the determinants of binding affinity and specificity for Late domain recognition are partially encoded in the residues flanking the PPxY core motif in the viral proteins.

Binding of all ligands to hNEDD4-WW3 show the thermodynamic profile previously described for polyproline recognition by other WW and SH3 domains: i.e. the interaction is driven by markedly favorable binding enthalpies partially opposed by unfavorable entropic contributions. The binding enthalpy of the isolated PPPY motif represents between 55% to 75% of the binding enthalpy of the different ligands (Table 1 and Fig. 1), indicating that, even though the establishment of additional interactions with flanking residues in the peptide ligands might modulate the magnitude of the enthalpic contributions, the exothermic character of the interaction is inherent to the recognition of the proline-rich PPxY motif. The Ebola ligand presents an enthalpy change of −50.7 kJ·mol^−1^, very similar to the nonspecific high affinity ligand p53bp2 and only slightly higher than the isolated PPPY motif. Nonetheless, all other Late domain peptides are characterized by more exothermic binding enthalpies, close to −70 kJ·mol^−1^. Even though the impact of this large enthalpic difference (20 kJ·mol^−1^) on the binding affinity is mitigated by enthalpy/entropy compensation effects, it reflects a modulation in the balance of forces driving the interaction for the two groups of ligands (p53bp2, PPPY and Ebola peptides vs HTLV1, Rabies and Marburg sequences), suggesting differences in the way they interact with the hNEDD4-WW3 domain.

### NMR structural characterization of hNEDD4-WW3/Late domain complexes

**Figure 2:**
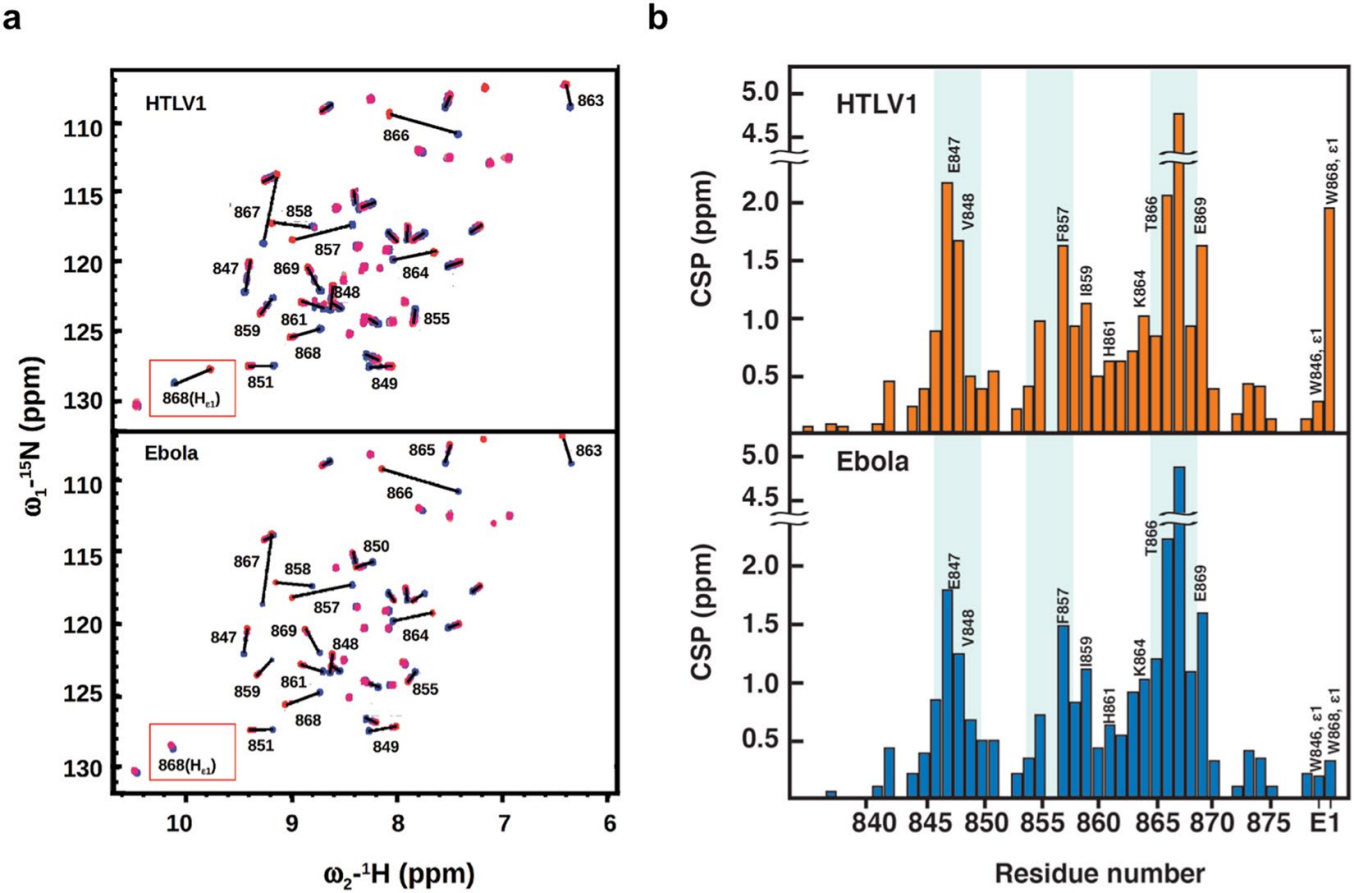
NMR titration of Ebola and HLTV1 Late domains onto the hNEDD4-WW3 domain. (**a**) Superposition of four ^1^H-^15^N-HSQC spectra of hNEDD4-WW3 domain at different protein/ligand ratios. Upper panel shows spectra obtained at 1:0.0 (blue), 1:0.5 (purple), 1:1.5 (pink) and 1:3.0 (orange) molar ratios for the HTLV1 complex. Lower panel shows spectra at 1:0.0 (blue), 1:1.0 (purple), 1:2.5 (pink) and 1:10.0 (orange) molar ratios for the Ebola complex. The signal of the indole proton of W868, which shows a differential behavior in the two complexes, has been highlighted in a red box. (**b**) Average Chemical Shift Perturbations (CSPs) calculated at the last titration point for each residue in the ^1^H-^15^N-HSQC spectra of NEDD4-WW3 domain in the presence of 1:3 excess HTLV1 peptide (upper panel) and 1:10 excess Ebola peptide (lower panel). The experimental error for the spectral resolution is estimated to be ±0.03 ppm (see Methods for details).

The Ebola and HTLV1 Late domain peptides were selected as examples of each group of ligands and ^1^H-^15^N-HSQC NMR titration experiments were performed to evaluate possible differences in hNEDD4-WW3 recognition. Addition of unlabeled HTLV1 and Ebola peptides to the previously assigned ^15^N-labeled NEDD4-WW3 domain resulted in significant chemical shift perturbations (CSP) of the backbone amide signals throughout the domain, eliciting a very similar pattern for both complexes (Fig. 2a). The largest perturbations were observed for residues defining the xP and xY canonical binding pockets^22^: F857, F858 and I859 (β_2_ strand); H861 and K864 (β_2_/β_3_ loop), T865, T866 and T867 (β_3_ strand); and W868 and E869 (Fig. 2b). Additionally, binding of HTLV1 and Ebola affects residues E847, V848 and R849 (β_1_ strand) and R855 (β_1_/β_2_ loop) outside the xP and xY pockets, in agreement with previous reports on other WW complexes^23–28^. In spite of the similarity between the two complexes, the indole group of W868 (H_ε1_) at the xP pocket, previously used to monitor binding in several WW domains^13,21^, responds differently to the presence of each ligand, experiencing a large shift in the HTLV1 complex (CSP ~ 2 ppm) that is much reduced for Ebola (CSP < 0.5 ppm).

To further investigate these differences, the structures of the HTLV1 and Ebola complexes were solved by NMR. A total of 50 (HTLV1) and 52 (Ebola) manually assigned intermolecular NOEs were used for the calculation of the complex structures, the most relevant of which are summarized in Supplementary Table S2. Most NOEs with the WW domain were concentrated in the PPxY core motif region, which adopts a PPII conformation and shows the typical binding mode described for other WW complex structures^23,27–29^. For both complexes, the first two prolines in the motif (P_−3′_ and P_−2′_) are involved in stacking interactions at the xP pocket of the domain (P_−3’_ shows several NOEs to the W868 aromatic ring and P_−2′_ interacts with the F857 side chain). Also, the tyrosine residue at the core motif (Y_0′_) shows NOEs to the methyl groups of T866, to the γ protons of I859 and K864, and to the α and ε protons of H861 in the two structures.

No NOE signals could be detected for the first three residues at the N-terminus of the HTLV1 and Ebola ligands (S_−8_D_−7_P_−6_ and I_−8_L_−7_P_−6_ respectively), suggesting lack of interaction with the hNEDD4-WW3 domain. To evaluate any possible influence of these residues, we calculated the structures of the full-length Ebola and HLTV1 complexes and their shortened versions (HLTV1: Q_−5_IP_−3_P_−2_PY_0_VEP_3_ and Ebola: T_−5_AP_−3_P_−2_EY_0_MEA_3_), which correspond to the part of the peptides with inter-molecular NOEs. As illustrated in Supplementary Fig. S1a, showing the superposition of the 20 lowest energy NMR ensembles obtained with the short and full-length peptides, the structural models are fully equivalent within experimental resolution. The main difference is observed at the first three N-terminal residues, which appear unstructured and randomly distributed for both HTLV1 and Ebola complexes. Moreover, the presence or absence of these three residues does not alter the side chain conformation of the well-defined regions of the ligand (Supplementary Fig. S1b), confirming that the complexes with the short peptides capture all essential elements of Ebola and HTLV1 Late domain interaction with hNEDD4-WW3.

This observation is in agreement with previous studies on NEDD4-WW3 complexes indicating that N terminal residues beyond position −5 do not influence NEDD4-WW3 recognition. For instance, Kanelis *et al*.^25^ found that elongating the T_−5_GL_−3_P_−2_SY_0_DEA_3_LH (K_d_ = 3.1 μM) peptide two positions N-terminal did not have any effect on binding affinity. Similarly, the R_−6_P_−7_ residues of the high affinity (K_d_ = 3 μM) ARRDC3 ligand (RPE_−5_AP_−3_P_−2_SY_0_AEVVT) were also not observed in the crystal structure of the complex (4N7H), probably because these residues populated several conformations^28^. All these observations led us to consider that NMR-based models calculated with the short versions of the Ebola and HTLV1 peptides recapitulate the hNEDD4-WW3 Late domain binding properties to the Ebola and HTLV1 peptides and these complexes were deposited in the PDB under the codes 2KQ0 and 2KPZ respectively.

**Figure 3:**
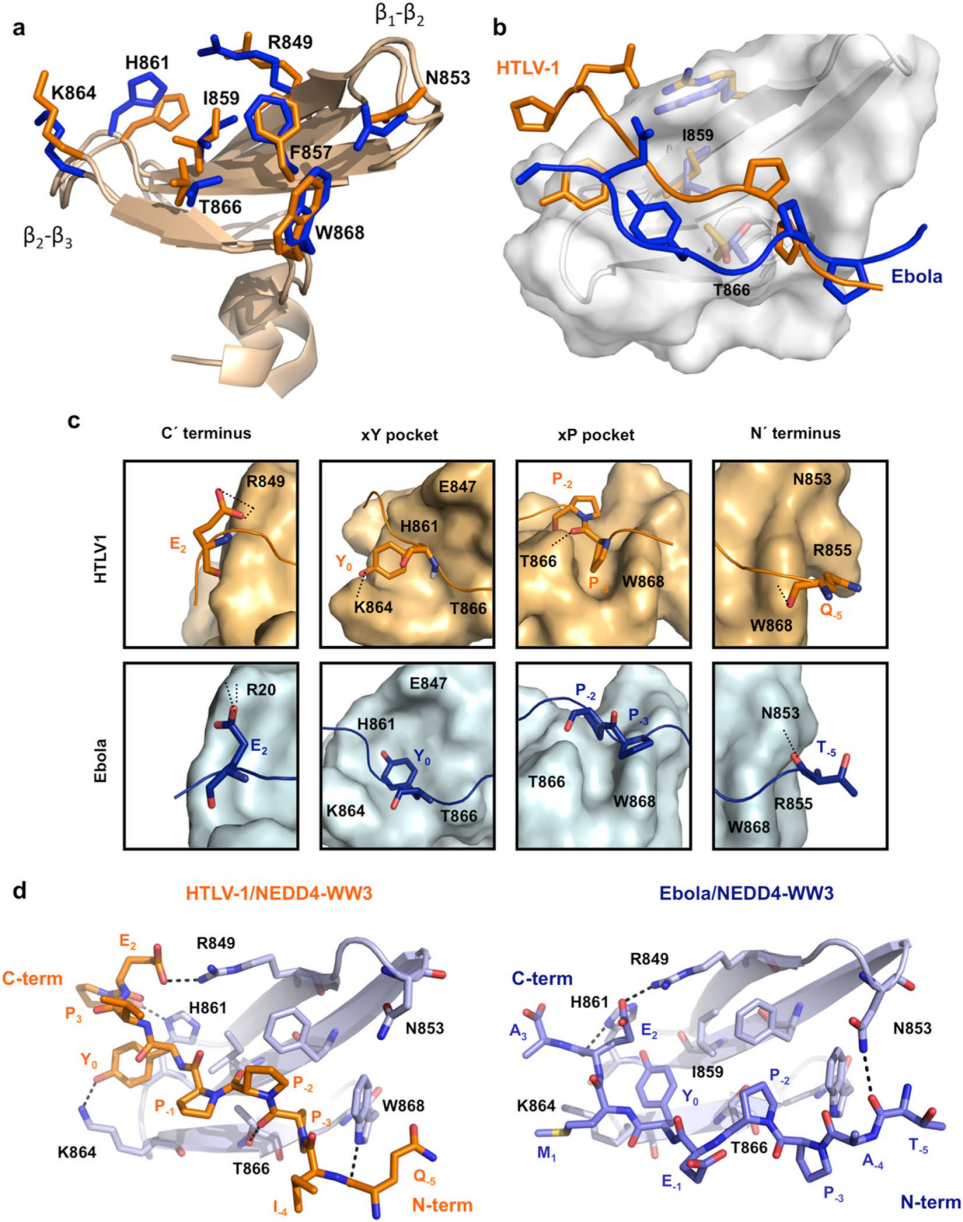
Structural comparison of the Ebola and HTLV1 complexes with hNEDD4-WW3. (**a**) Structural superposition of the hNEDD4-WW3 domain structure (light brown cartoons) from the Ebola and HTLV1 complexes. Shown are the lowest energy models in the NMR ensembles. The side chains of the WW domain residues involved in binding are shown as orange (HTLV1) and blue (Ebola) sticks. (**b**) Orientation of the Ebola (blue) and HTLV1 (orange) ligands on the hNEDD4-WW3 binding site. Residues I859 and T866, determining the size of the xP and xY pockets in these complexes and defining ligand orientation are shown as blue (Ebola) and yellow (HTLV) sticks. (**c**) Detail of the interactions established by the HTLV1 (upper panels) and Ebola (lower panels) ligands at the different regions on the hNEDD4-WW3 binding site. (**d**) Intermolecular hydrogen bonds established between the HTLV1 (left panel) and the Ebola (right panel) Late domain peptides and the hNEDD4-WW3 domain. The WW domain is shown in a light blue cartoon representation. The most relevant side chains for ligand recognition are shown in light blue sticks. Ligand atoms are shown as orange (HTLV1) and dark blue (Ebola) sticks. Hydrogen bonds are depicted as discontinuous black lines.

The twenty lowest energy structures after water refinement for each complex (Supplementary Fig. S2) show an average Root Mean Square Deviation (RMSD) for all backbone heavy atoms of 0.60 Å for the HTLV1 complex and 0.83 Å for the Ebola complex. The statistics of the calculation are summarized in Table 2. Most residues in both complexes were found in favored or allowed regions of the Ramachandran plot. In both structures the hNEDD4-WW3 domain presents the right hand twisted anti-parallel β sheet fold characteristic of WW domains, showing three well defined β strands comprising residues E847-A851 (β_1_), P856-D860 (β_2_), and T865-T867 (β_3_) (Fig. 3a). Overall, the backbone structure of the WW domain does not differ between the HTLV1 and Ebola complexes and is also very similar to other hNEDD4-WW3 structures (Supplementary Fig. S3).

**Table 2:** Structural statistics for the NEDD4-WW3/Late domain complexes.

	HTLV1 PDB:2KPZ	Ebola PDB: 2KQ0
**Number of structural restraints**		
**All restraints**	**679**	**625**
Sequential (|i − j| = 1)	259	236
Medium range (1 < |i − j| ≤ 4)	90	72
Long range (|i − j| > 4)	264	209
**Intermolecular**	**50**	**52**
Ambiguous restraints	0	0
**Dihedral angles**	**50**	**40**
**Hydrogen bonds**	**16**	**16**
Average restraints per residue (43 residues)	15.7	14.5
**RMSD from the restraints** ^**a**^ **<SA>** ^**b**^		
All NOE distance restraints (Å):	10.1·10^−3^ ± 3·10^−4^	4.4·10^−3^ ± 4·10^−4^
Bonds (Å)	8.3·10^−3^ ± 2·10^−4^	4.2·10^−3^ ± 2·10^−4^
Dihedral angles (°)	0.83 ± 0.03	0.59 ± 0.02
**Average atomic RMSD from the minimum energy structure** (**Å**), (**whole complex**)		
Backbone N, C_α_ and CO atoms, (secondary structure only)	0.43	0.59
Backbone N, C_α_ and CO atoms all residues	1.15	1.56
All Heavy atoms, all residues	2.48	2.66
**Average atomic RMSD from the complex** (**Å**), (**domain only in the complex**)		
Backbone N, C_α_ and CO atoms, all residues	1.01	1.44
All Heavy atoms, all residues	2.47	2.54
**Average atomic RMSD from the complex** (**Å**), (**peptide only on the complex**)		
Backbone N, C_α_ and CO atoms, all residues	1.51	1.90
All Heavy atoms, all residues	2.65	3.24
**Energetic quality (kcal mol** ^**−1**^ **)**		
Total energy^c^	−1260 ± 50	−1540 ± 30
Electrostatic	−1630 ± 50	−1750 ± 40
van der Waals	−120 ± 10	−140 ± 10
Bonds	51 ± 2	13 ± 1
Angles	140 ± 10	72 ± 6
**Ramachandran map analysis**^**d**^ (**%**)		
Residues in most favoured regions	91.2	84.7
Residues in additionally allowed regions	7.3	14.1
Residues in generously allowed regions	1.5	0.9
Residues in disallowed regions	0	0.3

^a^After water refinement no distance restraint was violated by more than 0.3 Å.

^b^<SA> refers to the ensemble of the twenty structures with lowest energy.

^c^The total energy is the Lennard-Jones van der Waals energy (E_L-J_) calculated using CHARMMPARMALLH6 parameters^54^. E_L-J_ was not included in the target function during structure calculation. ^d^The Ramachandran map was calculated using PROCHECK^55^.

Comparison with the NMR structure of the ENaC complex (2M30^23^) and the crystal structure of the ARRDC3 complex (4N7H^28^) results in average RMSD values for the backbone heavy atoms that range between 1.0 and 1.2 Å. Comparison with the NMR (5AHT^30^) and crystal (4N7F^28^) structures of the free domain renders similar results, although RMSD values up to 1.8 Å are locally observed for residues A851, P852, N853 and G854 at the β_1_-β_2_ loop.

The side chains that constitute the hydrophobic buckle stabilizing the WW domain structure (W846, F858 and P871) and most residues implicated in ligand recognition (F857, H861, K864, and W868) also show very similar conformations in the Ebola and HTLV1 complexes (Fig. 3a). Nonetheless, residues I859 and T866, placed at the center of the binding site and delimiting the xP and xY pockets, are found in different conformers in each complex structure (χ1_I859_: −46° and χ1_T866_: +41° for HTLV1 and χ1_I859_: +43° and χ1_T866_: −18° for Ebola). These conformational differences, reminiscent of those observed between the crystal structure of the ARRDC3/NEDD4-WW3 complex and the free domain^28^, modulate the shape and size of the xP and xY pockets, inducing changes in the pattern of binding site interactions and a different orientation of the HTLV1 and Ebola ligands on the surface of the WW domain (Fig. 3b).

As illustrated in Fig. 3c, the HTLV1 rotamer combination results in a deeper xP pocket that allows a tight fit of the P_−2′_ and P_−3′_ side chains: a close contact between the HTLV1 P_−3′_ side chain and W868 is confirmed by the large chemical shift displacement of its indole amide upon ligand binding (Fig. 2); also, a direct hydrogen bond is formed between the T866 side chain and the carbonyl oxygen of P_−3′_ (Fig. 3d). These interactions are not observed in the Ebola complex, in which the conformation adopted by T866 reduces the size of the xP pocket, hindering the docking of P_−2′_ and P_−3′_, and placing the hydroxyl oxygen at distances incompatible with the formation of hydrogen bonding interactions with the carbonyl oxygen of P_−2′_ or P_−3′_. In compensation, the I859/T866 side chain arrangement in the Ebola structure results in a well-defined xY pocket that allows the tyrosine side chain in the PPxY motif (Y_0′_) to be deeply buried. A wider and shallower xY pocket is observed in the HTLV1 complex, characterized by a higher exposure of the Y_0′_ side chain, associated in part to the change in conformation of the H861 side chain, which is directed outwards in this structure (Fig. 3c and Supplementary Fig. S3). This is in agreement with the differences in the CSP values (1.2 ppm in Ebola vs 0.8 ppm in HTLV) registered for the backbone amide of T865 located at the bottom of the xY pocket (Fig. 2a).

Additional differences are also found at the N- and C-terminal regions of the ligands. As is common in viral Late-domains sequences, which frequently carry negatively charged residues one or two positions C-terminal from the PPxY motif^5^, both HTLV1 and Ebola ligands have a glutamate residue at position +2 that is implicated in electrostatic interactions with R849 at the β1 strand. Nonetheless, the assigned signals defining this interaction are also slightly different in the two complexes: the two NOEs observed between the ε protons of R849 and the γ1 and γ2 protons of E_2’_ in the HTLV1 ligand shift to the γ1 and β2 protons in the Ebola complex, resulting in a different conformation for the E_2′_ side chain (Fig. 3c). At the N-terminal region, weak NOEs signals are observed between residues at positions 5′ and 4′ in the ligand (HTLV1 Q_−5′_and I_−4′_ and Ebola T_−5′_ and A_−4′_) and the side chain of W868 at the end of the β3 strand (Supplementary Table S2). Nonetheless, the carbonyl oxygen of the residue at position −5′ forms an intermolecular hydrogen bond with different partners in each complex: HTLV1 Q_−5′_ interacts with the side chain of W868, establishing a hydrogen bond very similar to that found in the x-ray structure of the ARRDC3 complex^28^, while Ebola T_−5′_ is removed from the W868 side chain and found at hydrogen bond distance of the carboxyamide of N853 (Fig. 3d). This interaction brings the ligand closer to the β1-β2 loop that adopts a slightly different conformation in the two complex structures (Fig. 3a).

In summary, the structural analysis confirms small conformational differences at the binding site region and a different pattern of binding site interactions between the two complexes that correlate well with the thermodynamic results. In this way, a higher number of intermolecular interactions are established in the HTLV1 complex in comparison with the Ebola structure (Fig. 3d and Supplementary Table S3), in agreement with the more exothermic binding enthalpy of HTLV1 (ΔΔH_HTLV-Ebola_ = −20 kJ/mol^−1^). By extrapolation, the sign and magnitude of the binding enthalpies of the Rabies and Marburg Late domain suggest an HTLV1-like binding mode.

### Conformational properties of hNEDD4-WW3/Late domain complexes studied by Molecular Dynamics simulations

In order to further investigate the conformational variability observed for the hNEDD4-WW3 binding site, we calculated 40 ns molecular dynamics trajectories using our Ebola (2KP0) and HTLV1 (2KPZ) complex structures and the crystal structure of the free NEDD4-WW3 domain (4N7F) as starting points. For comparison, equivalent simulations were performed for the NMR structure of the apo mNEDD4-WW3 domain (1WR7), of very similar sequence and longer N- and C-termini (Supplementary Fig. S4a). The backbone root-mean-square deviation (RMSD) for the WW domain rapidly reaches a stable plateau at about 1–2 Å without abrupt transitions in the energy profile, indicating that in all cases the WW domain is stable throughout the simulations. A similar situation was found for the ligand atoms (Supplementary Fig. S4b).

Analysis of the average mass-weighted fluctuation per residue (Supplementary Fig. S4c) reveals that the β1-β2 and β2-β3 loop regions undergo large motions (2–2.4 Å) in the free domain simulations that are reduced upon ligand binding. Nonetheless, while the stabilization of the β1-β2 loop is very similar for both complexes, the β2-β3 loop remains more flexible in the HTLV1 complex. This structure presents a binding site geometry and ligand configuration very similar to that of ENaC/NEDD4-WW3 complex (2M30) (Supplementary Fig. S5a), for which NMR dynamic studies showed that in the bound state the β2-β3 loop exists in equilibrium between two different backbone conformations^23^. In contrast, the Ebola ligand seems to be more efficient stabilizing this region, possibly due to the better packing of Y_0′_ side chain at the xY pocket that allows more extensive contacts between C-terminal region of the ligand and the β2–β3 loop (Supplementary Fig. S5b).

The most relevant intermolecular interactions between the ligands and the hNEDD4-WW3 domain remain stable throughout de MD trajectories, providing a description of the two complexes consistent with the NMR structures. The average ligand/domain contact distances (Supplementary Fig. S6a) closely reproduce the experimental pattern of intermolecular NOEs, capturing the P_−3′_/W868 and P_−2′_/F857 contacts, the different packing of Y_0′_ at the xY pocket (I859, H861, K864 and T866), with 1–1.5 Å smaller average contact distances for the Ebola complex, as well as other interactions implicating the N- and C- terminal regions of the ligands (W868 with residues at position −4′ and −5′ and R849 with E2′). The hydrogen bond pattern also reflects the differences between the two complexes (Supplementary Fig. S6b).

**Figure 4:**
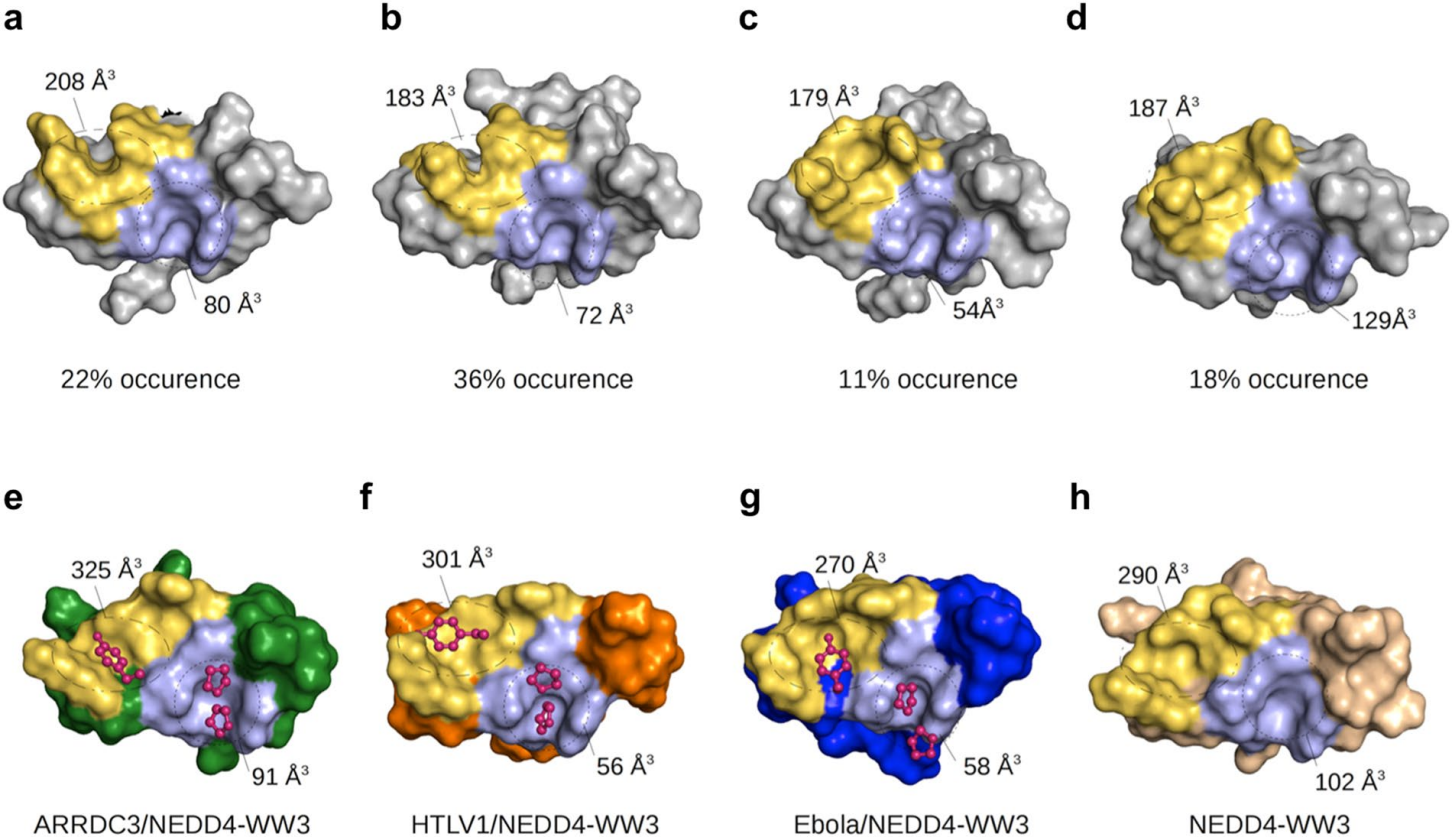
Variability in hNEDD4-WW3 binding site geometry. Uppers panels (a–d) show the surface depiction of the representative structures from the four most populated clusters in the apo-NEDD4-WW3 domain simulation. Lower panels show the surface representation of the hNEDD4-WW3 domain from the crystal structure of the ARRDC3 complex (4N7H)^28^ (**e**), the lowest energy NMR models for the HTLV1 and Ebola complexes (this work 2KPZ and 2KQ0) (**f**,**g**) and the crystal structure of the apo NEDD4-WW3 domain (4N7F) (**h**). Residues in the xY pockets are colored in yellow, the xP pocket in light blue. Shown are the estimated sizes for the two pockets calculated with FPocket^68^.

Globally, the simulations reveal a high level of binding site plasticity for the free NEDD4-WW3 domain that is drastically reduced upon binding of the two ligands. A cluster analysis of the two MD trajectories obtained for the free NEDD4-WW3 domain (4N7H and 1WR7) consistently produced four highly populated clusters, with occurrences ranging between 11% and 36% of the simulation time that show a considerable variability in the size and shape of the xP and xY pockets (Fig. 4a–d).

**Figure 5:**
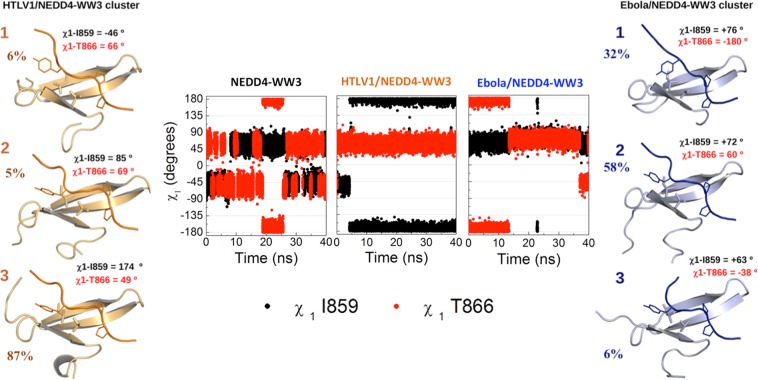
Molecular dynamics study of hNEDD4-WW3 conformational plasticity. Modulation of the hNEDD4-WW3 conformational distribution upon ligand binding. The central panel shows the evolution throughout the trajectory of the I859 (black dots) and T866 (red dots) side chain dihedrals for the apo domain and the Ebola and HTLV1 complexes. Right and left panels show representative structures of the most populated clusters for the Ebola (blue) and HTLV1 (orange) simulations. Also shown are the respective frequencies of occurrence and the side chain dihedrals for the I859 and T866 residues.

Binding of the HTLV1 and Ebola ligands results in narrower conformational distributions. Three clusters with occurrences >5% were identified for each complex, characterized by very similar backbone conformation for the WW domain (RMSD = 1.3 Å for the HTLV1 and 0.9 Å for the Ebola complex) but larger deviations for the ligand atoms (RMSD = 3.8 Å for HTLV1 and 3.5 Å for Ebola). The HTLV1 complex is characterized by a clearly predominant cluster that accounts for 87% of the simulation time while the other two clusters are only sporadically visited (occurrences of 6% and 5%). The Ebola complex retains a higher level of conformational flexibility at the binding site, showing two highly populated clusters, with 58% and 32% occurrence (Fig. 5).

Analysis of the conformations adopted by the Ile859 and Thr866 side chains in the MD trajectories (Fig. 5) parallels this behavior, highlighting the binding site plasticity of the apo domain and the conformational differences between the two complexes. The I859/T866 pair shows high side chain mobility in free domain, frequently fluctuating between different combinations of dihedrals: Ile859 alternates mostly between the two gauche rotamers while T866 shows more variability, visiting also the trans conformer. The presence of the two ligands induces a marked reduction in the mobility of these two residues, with each ligand preferentially populating a specific set of conformations: a clearly predominant combination of dihedrals, accounting for 85% of the simulation time, is found in the HTLV1 trajectory, in contrast with the Ebola complex, that shows a higher conformational variability for the Thr866 side chain. Interestingly, none of the χ1 combinations found for one complex are observed in the other, except for the most frequent conformation for the Ebola complex (cluster 2) that is only transiently observed for HTLV1 (5% occupancy). The predominant conformation for HTLV1 is not significantly populated in the Ebola trajectory.

**Figure 6:**
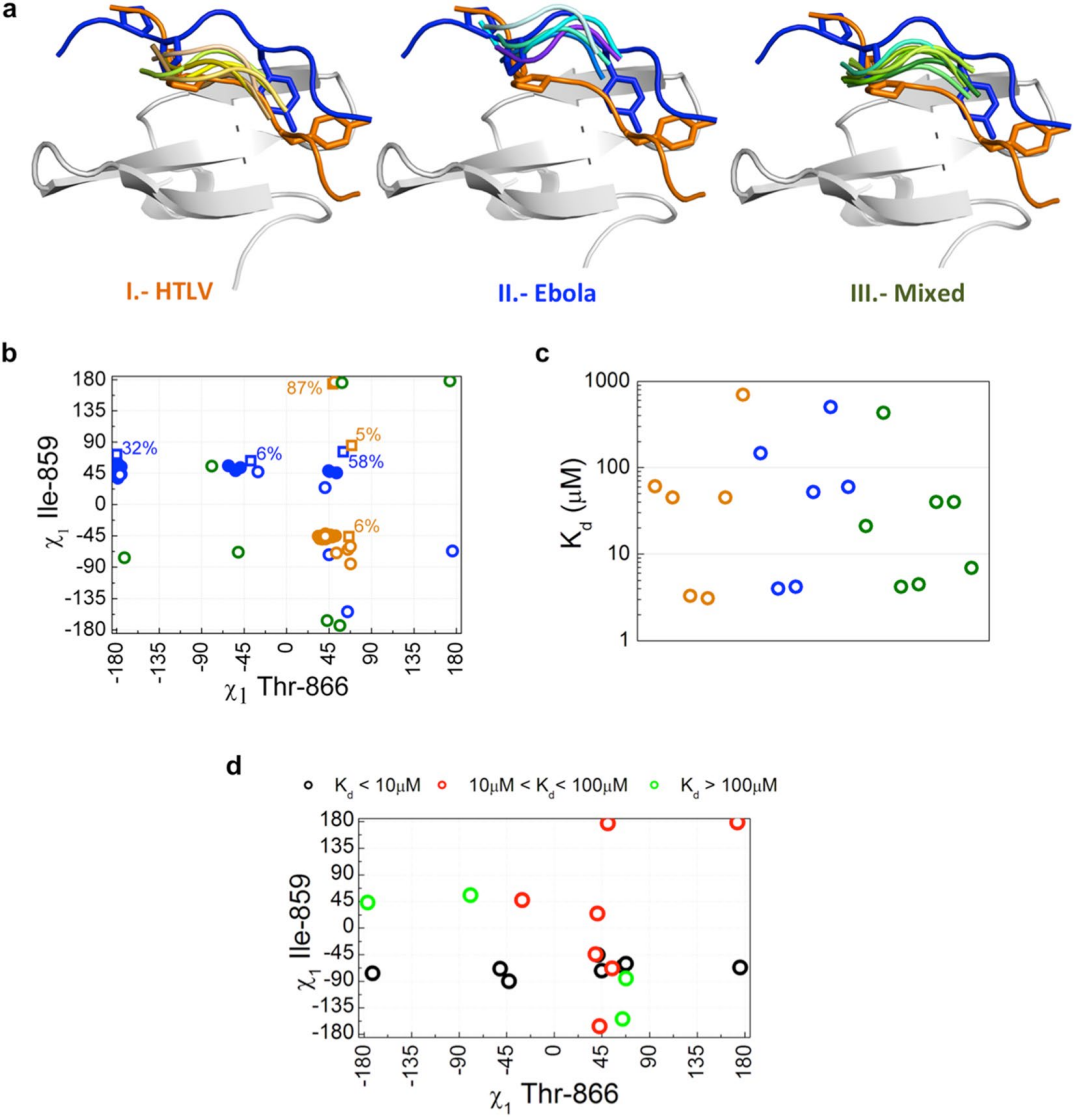
Variability in ligand binding orientation for type I WW domains. (**a**) Cartoon representation of the hNEDD4-WW3 domain (light gray). The Ebola and HTLV1 ligands are shown as blue and orange ribbons, with the Y_0_ and P_−2_, P_−3′_ side chains shown as sticks. The PPxY core motif regions of the different ligands classified in each category are shown as ribbons. (**b**) Side chain dihedral angles for Ile858 and Thr866 in type I WW domains complexes. Open circles represent the side chain conformations observed in the high-resolution structures showing HTLV1-like (orange), Ebola-like (blue) or mixed (green) orientations. Following the same color code, full circles show the conformations adopted in the 20 lowest energy models from the Ebola and HTLV1 NMR ensembles, and open squares the most populated clusters identified in the molecular dynamics trajectories. Numbers indicate frequencies of occurrence. (**c**) Dissociation constants for the WW complexes in the structural database (see Supplementary Table S3) color coded by type of orientation: HTLV1-like (orange), Ebola-like (blue) and Mixed (green). (**d**) Side chain dihedral angles for Ile859 and Thr866 in type I WW domains complexes color coded according to binding affinity: K_d_ < 10 μM (black), 10 μM < K_d_ < 100 μM (red), and K_d_ > 100 μM (green).

The MD conformational behavior is also in agreement with the rotamer variability found for the Ile859/Thr866 pair in the NMR ensembles of the Ebola and HTLV1 complexes: HTLV1 presents a single and well defined conformation (χ1_I859_ = −46º ± 2º and χ1_T866_ = 42º ± 5º) in all 20 models, while Ebola shows a higher conformational variability, so that the 20 lowest energy structures in the Ebola ensemble are grouped in three sets of conformations, with frequencies of 65% (χ1_I859_ = 48º ± 6º / χ1_T866_ = −179º ± 6º), 20% (χ1_I859_ = 52º ± 3º / χ1_T866_ = −55º ± 5º) and 15% (χ1_I859_ = 47º ± 2º / χ1_T866_ = 48º ± 5º), in good correspondence with the MD clusters (see also Fig. 6).

Thus, the molecular dynamics study further sustains the idea of a differential recognition of the two ligands by the NEDD4-WW3 domain, revealing a higher level of conformational diversity for the Ebola complex with respect to HTVL1, in agreement with the thermodynamic results, showing a smaller entropic penalty for the binding of the Ebola ligand (−TΔΔS_Ebola-HTLV1_ = 24 kJ/mol^−1^).

### Analysis of the structural database for type I WW domain complexes

In order to assess whether one of the two orientations observed for the Ebola and HTLV1 ligands represents a “canonical” or most common binding mode for PPxY peptides, the 21 high-resolution structures currently available for class-I WW complexes, covering a range of two orders of magnitude in binding affinity, were examined (see Supplementary Table S4). Even though the structures of these closely related WW domains are very similar (RMSD values with respect to the free domain (4N7F) are below 1.5 Å in all cases), a significant dispersion in the orientation of the ligands was found, paralleling the situation described for the Ebola and HTLV1 complexes. The complex structures could be classified in three different groups according to the disposition of the ligand at the binding site: (I) a set of 7 structures resembling HTLV1; (II) a set of 6 structures with a ligand orientation similar to the Ebola complex and, (III) a larger set of 11 structures showing an intermediate orientation, resembling the HTLV1 structure at the xP pocket and showing xY pockets similar to the Ebola complex (Fig. 6a).

The combinations of Ile859 and Thr866 dihedrals found in the high resolution structures (Fig. 6b), reproduce those of the most populated clusters identified in the molecular dynamics trajectories of the Ebola and HTLV ligands and the conformations observed in the NMR structural ensembles. In agreement with the simulations, the HTLV1-like conformation is restricted to a narrow interval of Thr866-χ1 values, around 50°, in combination with two intervals of Ile/Val859-χ1 values centered at −50° and 180°. A significantly higher conformational variability is found for the Ebola-like complexes in which Thr866 can be found in two predominant conformations corresponding to the most populated clusters in the MD trajectory: one with Thr866-χ1 values similar to HTLV1 and a second interval centered around 180°. In both cases a significant dispersion in the values of Ile/Val859-χ1 is observed. The third class of ligands shows a conformational dispersion similar to that of the Ebola-type complexes.

These results suggest a high degree of plasticity in the recognition of PPxY containing ligands that can be accommodated with a wide range of orientations with respect to the WW domain binding site, determined by the set of rotamers adopted by Thr866 and Ile/Val859 side chains. No correlation between ligand orientation and binding affinity was observed, so that examples of high (K_d_ < 5 μM), medium (10 < K_d_ < 100 μM) and low (K_d_ > 100 μM) binding affinity are found in similar proportions for the three types of ligand orientations (Fig. 6b). Taken together, these results sustain the idea that binding site plasticity is not limited to low affinity complexes (which could be somewhat expected), but is rather a general feature of PPxY recognition by WW domains. Our analysis also indicates that high affinity ligand binding in type I WW domain is invariably associated to a very specific conformer for Ile859 (<χ1> = 67° ± 4° for all complexes with Kd < 5 μM), while a high variability is accepted for the Thr866 side chain.

## Discussion

The thermodynamic study of the binding energetics of isolated viral Late domain sequences to hNEDD4-WW3, confirms that these peptide ligands, in the absence of other elements from the full-length protein, retain the ability to selectively recognize its host cellular target. All Late domain ligands present the thermodynamic signature previously described for other proline rich recognition modules, including SH3^31–33^, UEV^34,35^ and other WW domains^28,30,36^, which, as previously discussed, cannot be fully rationalized considering exclusively the direct contacts between the predominantly hydrophobic interacting surfaces^32,37–39^. For the hNEDD4-WW3 complexes, this is illustrated by the large discrepancies found between the measured binding enthalpies and the intrinsic binding enthalpies calculated from the analysis of the interacting surfaces^16^ (Supplementary Table S5), see Methods for details. These calculations, which typically predict binding enthalpies in different systems with an average error of 1.3 kJ· mol^−1^, render theoretical values of −21 and −18 kJ· mol^−1^ for the Ebola and HTLV1 complexes, which are 30 and 50 kJ· mol^−1^ smaller than the experimental enthalpies. Similar discrepancies are found for the ENaC and ARRDC3 complexes. Thus, the direct ligand/domain contacts can’t fully explain neither the absolute enthalpy values nor the large enthalpic differences between the ligands.

The origin of this characteristic thermodynamic behavior has been widely studied for SH3 domains and attributed to the interplay of several factors complementing the direct interfacial interactions: the establishment of extensive and partially conserved networks of water-mediated interactions at the binding interface detected in crystal structures^37,38^, the reorganization of the conformational distribution of peptide and domain upon binding^31,33^ and the modulation of the dynamic properties of the protein^40,41^. The extent to which these factors contribute to the binding energetics in WW domains remains to be evaluated. High resolution crystal structures are scarce and, although some interfacial water molecules mediating the interaction between the domain and peptide ligands have been identified in a few WW complexes, the interaction networks are not as extensive as those observed in SH3 complexes^39^. In our particular case, no buried water molecules are found in the only crystal structure available of a hNEDD4-WW3 complex^28^, suggesting a marginal contribution of water-mediated interactions to the binding energetics of Late domain sequences. Conversely, the conformational contributions are likely to play a much more relevant role in WW binding energetics, since the small size of WW domains seems to impose a dual role in folding and function for many residues. Several folding and unfolding studies of WW domains have been performed in the past. The folding mechanisms of these domains seem to depend on their specific sequences, and due to their complexity, different experiments revealed different folding scenarios^42^. We have recently established that wild type NEDD4-WW3, NEDD4-WW4, FBP11-WW1, FBP11-WW2 and YAP65-WW1(L30K) are characterized by a downhill folding equilibrium that entails high flexibility and broad native state ensembles that can be continuously modulated by changes in conditions or mutations^43,44^.

hNEDD4-WW3 is one of the most paradigmatic examples of WW downhill folding, showing the lowest folding barriers and the highest level of flexibility amongst the studied WW domains^44^. This conformational variability is captured by the molecular dynamics study of the hNEDD4-WW3 free domain that depicts a highly plastic binding site (Fig. 4a–d) and shows that binding of the Ebola and HTLV1 ligands selectively stabilize a small set of conformations, reducing the conformational variability within the complex distribution. This suggests a conformational selection mechanism^45,46^, according to which different ligands would preferentially stabilize those states with optimal binding sites geometries. This scenario is also fully compatible with previous reports proposing a coupled folding/binding equilibrium for this domain^28,30^. Several pieces of experimental evidence support this idea: (a) the binding of the isolated PPPY motif elicits a high percentage (between 55 and 75%) of the binding enthalpy measured for longer and tighter binding ligands, suggesting that a good part of the conformational reorganization is associated to the insertion of the core motif in the xP and xY conserved pockets, (b) the binding heat capacity of the p53bp2 ligand to NEDD4-WW3 is much larger than that of YAP-WW1 (ΔC_p,app_ = −1.59 kJ K^−1^ mol^−1^ and ΔC_p,app_ = −0.90 kJ K^−1^ mol^−1^, respectively). This can be attributed to a higher level of conformational fluctuations in the free hNEDD4-WW3, in agreement with the stronger temperature dependence of the native state heat capacity observed by Differential Scanning Calorimetry for this domain^44^; and (c) large differences (up to 20 kJ·mol^−1^) in the enthalpic and entropic contributions to the binding affinity are observed between the different peptide ligands that cannot be accounted for by differences in the direct interactions at the binding interface. Also, the structure-based calculations reveal that binding to hNEDD4-WW3 elicits large enthalpic contributions associated to conformational effects (Supplementary Table S5, see Methods for details) that vary strongly between the different ligands, with values for ΔH_conf_ ranging between −30 and −65 kJ·mol^−1^. For other proteins studied, ΔH_conf_ typically assumes common values for the different ligands with a small standard deviation (between 0,4 and 5.9 kJ·mol^−1^)^16^. The large variability observed for the different hNEDD4-WW3 complexes (<ΔH_conf_ >= −48 ± 14 kJ·mol^−1^) further sustains the idea that each ligand stabilizes enthalpically different conformations within the hNEDD4-WW3 conformational ensemble.

The fact that hNEDD4-WW3 complex structures sample the three binding modes identified within the type I WW structural database, further illustrates the conformational pliability of this domain that emerges as a key determinant of its functionality. Analysis of the available structural information reveals that the variability in ligand disposition and binding site organization seems to be a general feature for Type I WW domains, even within the subset of closely related WW domains from E3 ubiquitin ligases, and independently of the strength of the interaction. These results highlight the general plasticity of the WW domain binding sites and their ability to adapt to different ligand sequences, within the framework of a conformational selection mechanism that seems to be universal for WW domains. The finding that all high affinity WW complexes studied exquisitely select a specific I859 conformer, clearly illustrates this point, and is of interest for the definition of the optimal binding site geometry against which to tackle the design of high affinity ligands of potential biotechnological or therapeutic interest.

The results of the structural, thermodynamic and molecular dynamics study presented here provide valuable insight into the molecular basis of viral Late domain recognition by hNEDD4-WW3. We have shown that the determinants of binding affinity and specificity are in a good extent encoded within short Late domain sequences, even in the absence of additional elements from the viral proteins. These Late domains seem to have evolved to achieve a higher level of intrinsic specificity than ligands of cellular origin, for which contextual factors, such as the presence of other protein domains, the expression profile or subcellular localization seem to carry more weight. This paints a favorable scenario for drug design, since it indicates that a good level of binding affinity and specificity could be attained in these systems using relatively small molecules. Since the recognition of PPxY ligands by WW domains involves adaptable binding sites with variable geometries and entails strong enthalpy/entropy compensation effects, which are major roadblocks in the design process, the incorporation of strategies involving rational and non-rational and/or high-throughput approaches to the discovery process could be a potent complement to more traditional structure-based design approaches.

## Methods

### Protein expression and purification

The third WW domain of human NEDD4 (hNEDD4-WW3) comprising aminoacids 834–878 (UniProtKB/Swiss-Prot code P46934) was cloned as described elsewhere^43^. The first and the second WW domains of human YAP2 (YAP-WW1 and YAP-WW2), comprising aminoacids 165–209 and 228–271 respectively (UniProtKB/Swiss-Prot code Q7Z574) were cloned by GENEART AG and subcloned into pETM-30 vector (Protein Expression and Purification Core Facility, EMBL, Heidelberg, Germany). All unlabelled protein samples were expressed as N-terminal GST-His-tagged proteins. For NMR experiments, the gene encoding the hNEDD4-WW3 domain was subcloned into a pETM-11 vector (Protein Expression and Purification Core Facility, EMBL, Heidelberg, Germany) and over-expressed in *E*. *coli* BL21/DE3 cells, fused to a His-tag at the N-terminus. The pETM30 and pETM11 plasmids introduce a TEV protease restriction site between the WW domain and the GST and His tags, respectively.

Uniformly labeled ^15^N hNEDD4-WW3 was expressed by growing cells in M9 medium containing ^15^NH_4_Cl as the only nitrogen source. For the production of the uniformly ^13^C- and ^15^N-labeled sample a minimal medium was used with [^13^C_6_]-glucose and ^15^NH_4_Cl as the only carbon and nitrogen sources, respectively. All protein samples were initially purified by nickel affinity chromatography, as described before^43^. GST and His-tag regions were removed by controlled hydrolysis with TEV protease. The isolated WW domains were further purified using a second step of nickel affinity chromatography followed by a gel filtration step on a HiLoad Superdex 75 column (GE healthcare Life Science). The purity and integrity of the purified proteins were checked by SDS-Page and Mass Spectrometry (Mass Spectrometry Service of the CIC at the University of Granada) and estimated to be over 99%. Immediately after the purification, all samples were frozen in liquid nitrogen in the purification buffer (300 mM NaCl 50 mM sodium phosphate buffer, pH 8.0) and stored at −80 °C.

### Peptide and protein samples

Protein concentrations were determined by absorption measurements at 280 nm using molecular weights of 5568 Da, 5524 Da and 5410 Da and extinction coefficients of 11380 cm^−1^·M^−1^, 12550 cm^−1^·M^−1^ and 13960 cm^−1^·M^−1^ determined as described by Gill & von Hippel^47^, for hNEDD4-WW3, YAP-WW1 and YAP-WW2, respectively. Synthetic lyophilized peptide ligands were purchased from SynBioSci Corporation with the exception of peptides Marburg and Rabies, which were bought from Peptide2.0 Corporation. All the peptides were acetylated and amydated at their N and C termini, respectively. They were synthesized on solid phase and their molecular mass was confirmed by mass spectrometry. Peptide purity (>95%) was assessed by analytical HPLC. For those ligands without tryptophan residues in their sequence (PPPY, p53bp2, HTLV1, Ebola and Marburg) peptide concentrations were determined by absorbance at 276 nm using an extinction coefficient of 1450 M^−1^ cm^−1^ per Tyrosine residue. Rabies concentration was measured by absorbance at 280 nm using an extinction coefficient of 6990 M^−1^ cm^−1^.

### Nuclear magnetic resonance (NMR) spectroscopy

hNEDD4-WW3 labelled samples were prepared by concentrating the protein in 300 μL or 600 μL of buffer 20 mM phosphate pH 7 in H_2_O:D_2_O (19:1 ratio by volume), containing 50 μM 2,2-dimethyl-2-silapentane-5-sulfonate sodium salt (DSS) as the internal reference for ^1^H chemical shifts. NMR experiments were performed at 288 K on a Bruker AV 700 spectrometer or on a Bruker AV 600 spectrometer equipped with a cryoprobe. The protein:peptide ratios for HTLV1 or Ebola peptides were 1:3 and 1:10, respectively, which correspond to the last addition of ligand used in the NMR titrations. For sequence-specific polypeptide backbone and side-chain chemical shift assignments ^1^H-^1^H TOCSY, ^1^H-^1^H NOESY, ^1^H-^15^N HSQC experiments were performed for both hNEDD4-WW3/HTLV1 and hNEDD4-WW3/Ebola complexes. Additional ^13^C-^1^H HSQC, HNCO, HNCACB, CBCA(CO)NH, (H)CC(CO)NH, ^15^N-^1^H TOCSY-HSQC and ^15^N-edited NOESY-HSQC were performed for the assignment of the HTLV1 complex^48^. Distance restraints were collected from the homonuclear and ^15^N-edited NOESY spectra recorded with a mixing time of 150 ms. Backbone ^1^H and ^15^N chemical shift assignments of the NEDD4-WW3 complexes were made using ^15^N-^1^H HSQC and ^15^N-edited NOESY-HSQC (80 ms mixing time) spectra recorded on uniformly ^15^N-labeled NEDD4-WW3 at 288 K. Chemical shifts were referenced to DSS used as an internal reference for ^1^H, and were calculated for ^15^N and ^13^C^49^. The average chemical shift perturbations resulting from complex formation were calculated using the equation:

$$\sqrt{{(\Delta N)}^{2}+{(5\cdot \Delta H)}^{2}}$$

in which ΔΝ and ΔΗ are the chemical shift changes in the ^15^N and ^1^H resonances, respectively. The threshold for significant changes (0.03 ppm) was calculated using the spectral resolutions in the ^1^H and ^15^N acquisition dimensions of the HSQC spectra as the values for Δδ_H_ and Δδ_N_, respectively.

### Structure calculations

Peak lists for the NOESY spectra were generated by interactive peak picking, and peak volumes were determined by the automatic integration function of XEASY package using the maximum option^50^. All data were processed using NMRPipe/NMRDraw^51^ and analyzed with CARA. Manually unambiguosly assigned peaks, dihedral angle restraints obtained from the backbone chemical shifts using TALOS^52^ and hydrogen-bond for antiparell strands were used during the calculation. The structures were calculated employing CNS^53^ with an in-house modified protocol of Aria1.2^54^. The protocol calculates a set of 120 structures, with 100,000 cooling steps according to Morales *et al*.^27^. The structural quality of the final 20 structures was evaluated with PROCHECK-NMR^55^, which also provided an average structure of the NMR ensemble. The statistics derived from the analysis are shown in Table 2. The resonance assignments have been deposited with BMRB entries 16574 and 16575 and the refined models have been deposited with PDB entries 2KPZ and 2KQ0 for the HTLV1 complex and for the Ebola complex respectively.

The WW domain in the bound state was assigned using standard triple resonance experiments. The complete sequences of HTLV1 and Ebola ligands (Supplementary Table S1) bound to the WW3 domain were assigned using 2D ^1^H-^1^H TOCSY and ^1^H-^1^H NOESY experiments. All structure calculations were performed with N-term acetylated and C-term amidated peptides. Because only some ligand residues produced intermolecular NOE signals in the presence of the domain, shorter peptides sequences (2′-QIPPPYVEP-10′ for HTLV1 and 2′-TAPPEYMEA-9′ for Ebola) were considered for the final calculation of the complex structures. The structural restrains for the secondary structure were obtained by the conformational relationships by residue based on the difference between the experimental and theoretical unfolded values of each nuclei^49^ and by the assignment of medium-distance and inter-stranded NOE signals.

### Fluorescence spectroscopy

The fluorescence emission spectra of the tryptophan residues in the WW domains were used to monitor any change in their environment upon peptide binding. Fluorescence was measured in an Eclipse spectrofluorimeter (Cary Varian). Samples were excited at 298 nm with a 5 nm slit and the fluorescence emission was detected between 300 nm and 500 nm through a 5 nm slit. Experiments were performed in 20 mM sodium phosphate pH 7.0, 25 °C at a protein concentration of 20 μM in a 1 cm path length cuvette. Each spectrum was corrected for ligand intrinsic fluorescence and normalized for protein concentration. Changes in spectral area due to intensity changes and shifts of the maximum of emission, were observed during the titration. The center of spectral mass was calculated as^56^:

$${\boldsymbol{\nu }}=\sum {\nu }_{i}{F}_{i,N}/{F}_{i,N}$$

Where ν is the center of mass in wave numbers, *F*_*i*_,_*N*_ is the normalized fluorescence emitted at wave number ν_*i*_. Assuming a one to one complex between the WW domains and the peptide, the *K*_d_ for the interaction of the different peptides was determined by fitting the changes in the CM versus ligand concentration, L_T_, to the equation:

$${\boldsymbol{\nu }}={\nu }_{M}+({\nu }_{ML}-{\nu }_{M})\cdot \frac{({M}_{T}+{L}_{T}+{K}_{d})-\sqrt{{({M}_{T}+{L}_{T}+{K}_{d})}^{2}-4{M}_{T}{L}_{T}}}{2{M}_{T}}$$

Where ν_M_ is the center of mass for the free domain spectrum, ν_ML_ is the center of mass for the spectrum of the complex (the last spectrum of the titration) and Μ_T_ is the total concentration of the domain in the experiment.

### Isothermal titration calorimetry (ITC)

ITC experiments were performed using a high precision VP-ITC titration calorimetric system (Microcal Inc., Northampton, MA). The WW domains were extensively dialyzed against the titration buffer (20 mM sodium phosphate at pH 7.0). All solutions were filtered, properly degassed to avoid bubble formation, and equilibrated to 25 °C prior to each experiment. The protein solution (at 40–65 μM) in the calorimetric cell was titrated directly with the appropriate ligand (at 600–800 μM) dissolved in the dialysis buffer following a profile of injection volumes from 3 to 20 μl to better define the titration curve. The heat evolved after each peptide injection was obtained from the integral of the calorimetric signal. The heat produced by the binding reaction between the WW domains and the peptides was obtained as the difference between the heat of reaction and the corresponding heat of dilution, as obtained from independent titrations of the peptides into the buffer. The resulting binding isotherms were analyzed by nonlinear least square fittings of the experimental data to a model corresponding to a single set of identical sites, as previously described^32^. For those ligands with binding affinities out of the range measurable directly by ITC^57^ (ligands PPPY, Ebola-ter and HTLV1-ter peptides with NEDD4-WW3 and YAP-WW1 domains) displacement experiments were performed^58^ using the p53bp2 peptide as a competing ligand. In these cases, the protein solution (ca. 55 μM) with the peptide of interest at a molar ratio of saturation (ca. 0.8 mM) was placed in the calorimetric cell and titrated with the p53bp2 peptide (at 2 mM) following a profile of injection volumes from 4 to 20 μl. The resulting binding isotherms, corrected for the dilution heats, were analyzed using the ORIGIN 7 software (Microcal Inc., Northampton, MA, USA) according to the Sigurskjold displacement model^59^. Typically, the variability in the experimental values were estimated to be about 1% in the number of binding sites, 5% in the binding enthalpy and 10% in the binding affinity.

### Molecular dynamic simulations (MD)

We used the atomic coordinates of the HTLV1/NEDD4-WW3 and Ebola/NEDD4-WW3 determined in this work by NMR (see description above) as a starting point. The ligands were acetylated and amidated in their extremes to avoid unrealistic electrostatic interactions using PyMOL Molecular Graphics System (Version 1.5.0.4 Schrödinger, LLC). Computation of the protonation state of ionisable groups was performed with pdb2pqr^60^. Counter-ions (to maintain system neutrality) and explicit water molecules in a truncated octahedron box of TIP3P waters with 15 Å of cut-off were added using the LEAP module of AMBER 12^61^. Long range electrostatic interactions were treated by Particle-Mesh Ewald (PME)^62^. The standard ff12SB force filed was used to compute the interactions within protein^63^. The SHAKE algorithm^64^ was employed to constrain all bonds including hydrogen atoms. The systems were relaxed by three solvent minimization steps (5000-step steepest descent and 500-conjugate gradient steps) to remove structural clashes, followed by 20 ps heating up to 300 K and allowed to equilibrate during 100 ps following 5 steps progressively reducing restrictive restrains, until the absence of restrains in the last equilibration step. 40 ns of productive MD runs were performed in periodic boundary conditions in an isothermal isobaric ensemble at 1 atm, with 2-fs time integration steps. The trajectories were analyzed with the ptraj module of AMBERtools 12.0^61^ and the free energies of binding were estimated with the MM-ISMSA approach^65^. A total of 10 clusters per trajectory were generated using the AMBER cpptraj command. Clusters with occurrences below 3% were considered as non-significantly populated.

### Structure-based thermodynamic calculations

The analysis of the intermolecular interactions in the complex structures was performed using YASARA Structure^66^ with the standard parameters and definitions. Specifically, hydrogen bonds between potential donors and acceptors are considered if the hydrogen bond energy is better than 6,25 kJ·mol-1, which is 25% of the optimum value of 25 kJ·mol-1. Hydrogen bond energy is calculated according to the following expression:

$$Energ{y}_{Hbond}=25\cdot \frac{2.6-\,max(Di{s}_{H-A},\,2.1)}{0.5}\bullet Scal{e}_{D-H-A}\bullet Scal{e}_{H-A-X},$$

where Dis_H-A_ is the Hydrogen-Acceptor distance in Å; Scale_D-H-A_ is a scaling factor dependent on the angle formed by donor-hydrogen-acceptor; and Scale_H-A-X_ is a scaling factor dependent on the angle formed by hydrogen-acceptor and an atom, X, covalently bound to the acceptor.

The intrinsic binding enthalpy values were calculated according to Freire’s structural parameterization of the energetics^67^, as previously described^16^, using the lowest energy models of the Ebola (2KQ0), HTLV1 (2KPZ) and ENaC (1M30) NMR complexes and the x-ray structure of the ARRDC3 complex (4N7H). The intrinsic binding enthalpy was calculated according to the equation:

$$\begin{array}{c}\Delta {H}_{binding}(25{}^{\circ }C)=\varDelta {H}_{conf}(25{}^{\circ }C)+\varDelta {H}_{int}(25{}^{\circ }C)\\ \,\,\,\,\,\,\,\,=\Delta {H}_{conf}(25{}^{\circ }C){\textstyle \text{-}}7.35\cdot \varDelta AS{A}_{ap}+31.06\cdot \varDelta AS{A}_{pol}\end{array}$$

Where *ΔH*_*int*_ is the intrinsic binding enthalpy associated to the direct contacts between the protein domain and the ligand, *ΔH*_*conf*_ correspond to the enthalpic contributions associated to changes in the conformational distributions of ligand and/or protein, *ΔASA*_*ap*_ and *ΔASA*_*pol*_ are the changes in accessible surface area upon binding of the ligand calculated according to the Lee and Richards algorithm as the average of 64 different ligand orientations and a probe radius of 1.4 Å.

### Accession numbers

The structures solved in this work have been deposited in the Protein Data Bank with the PDB-ID 2KQ0 (Ebola/hNEDD4WW3) and 2KPZ (HTLV1/hNEDD4-WW3).

## Supplementary information

Supplementary video 1

Supplementary video 1

Supplementary video 1

Supplementary Information
