# Supplementary material for: Binding site plasticity in viral PPxY Late domain recognition by the third WW domain of human NEDD4
Source: Sci Rep. 2019 Oct 21;9:15076. doi: 10.1038/s41598-019-50701-3 (PMC6803667; doi:10.1038/s41598-019-50701-3)
Supplement: Supplementary file 4 — Supplementary Information [file 41598_2019_50701_MOESM4_ESM.pdf]

# Supplementary Information

# Plasticity in viral PPxY Late domain recognition by the third WW domain of human NEDD4

Manuel Iglesias-Bexiga[a,1], Andrés Palencia[a,2], Carles Corbi-Verge[b], Pau Martin[c], Francisco J. Blanco[d,e], Maria J. Macias[c,f], Eva S. Cobos[a] and Irene Luque[a*]

[a]Department of Physical Chemistry and Institute of Biotechnology. University of Granada, 18071, Granada, Spain.

[b]Donnelly Centre for Cellular and Biomolecular Research, Faculty of Medicine, University of Toronto, Toronto, Ontario, Canada.

[c]Institute for Research in Biomedicine (IRB Barcelona), The Barcelona Institute of Science and Technology (BIST). Baldiri Reixac, 10, Barcelona 08028, Spain.

[d]Structural Biology Unit, CIC bioGUNE, Parque Tecnológico de Bizkaia, 48160 Derio, Spain.

[e]IKERBASQUE, Basque Foundation for Science, María Díaz de Haro 3, 48013 Bilbao

[f]ICREA, Passeig Lluís Companys 23, 08010-Barcelona, Spain.

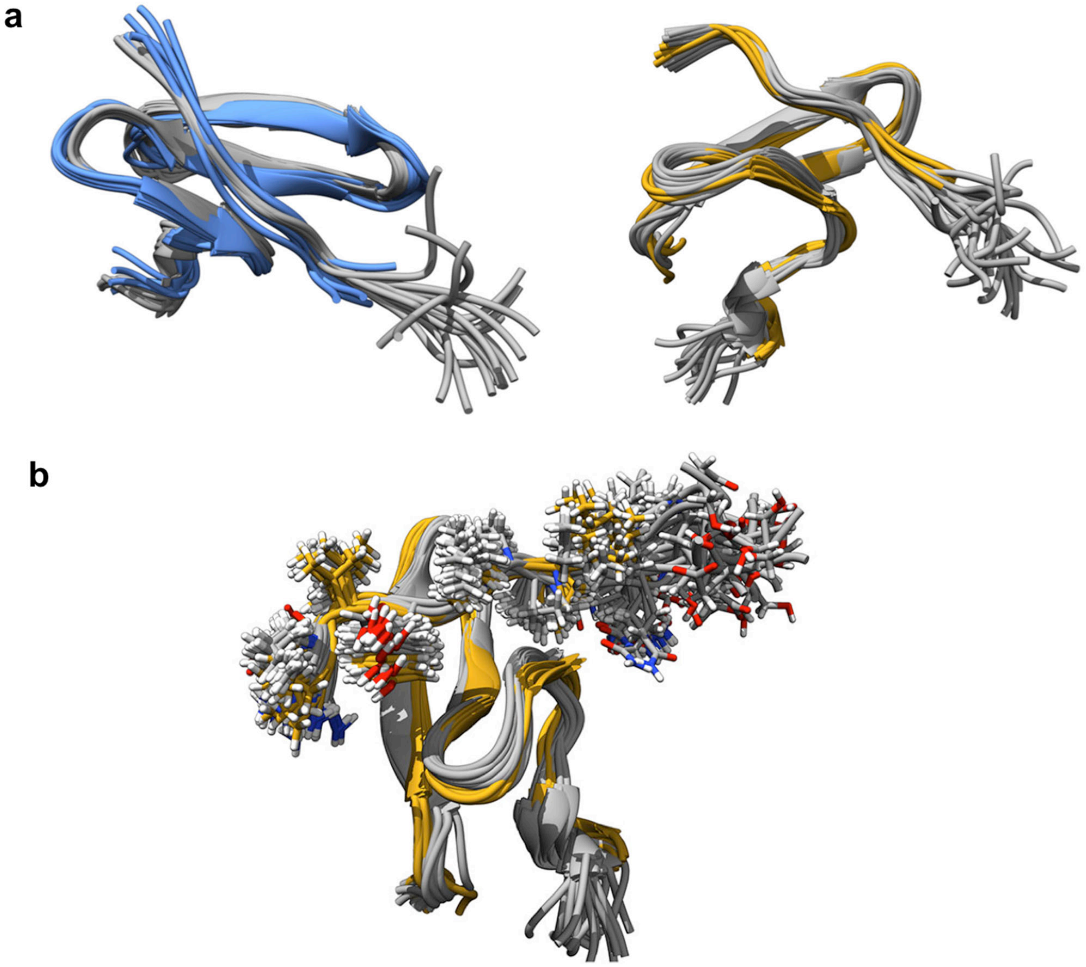


**Supplementary Figure S1. Impact of ligand length on the NMR structural calculation of Ebola/NEDD4-WW3 and HTLV1/NEDD4-WW3 complexes. (a)** Shown are the NMR ensembles of the 20 lowest energy models calculated for the Ebola (left panel) and HLTV1 (right panel) complexes. Structures corresponding to the full-length peptides are shown as grey cartoons while those corresponding to the shorter versions of the peptide ligands are shown in orange for HTLV1 and blue for Ebola. **(b)** Effect of changing peptide length of the side chain conformations of the ligand in the HTLV1/NEDD4-WW3 complex.

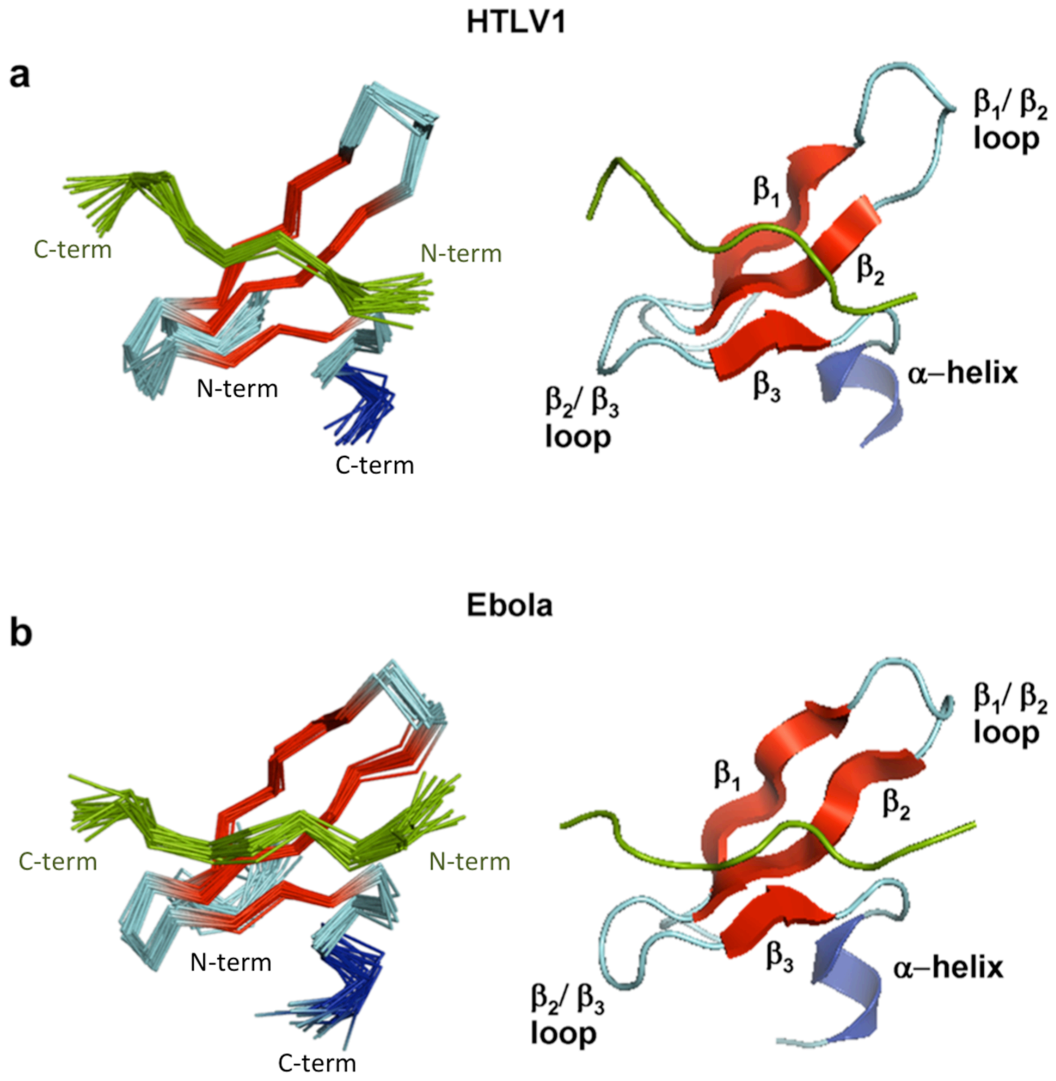


**Supplementary Figure S2**. **Solution structures of the NEDD4-WW3 domain in complex with the HTLV1 (a) and Ebola (b) Late domains**. Ribbon representation of the twenty lowest-energy structures after water refinement. The two representations show the complexes in the same orientation. The ligands are shown in green and the WW domain regions corresponding to the β strands are indicated in red, the loops in cyan and the α-helix of the C terminus in blue.

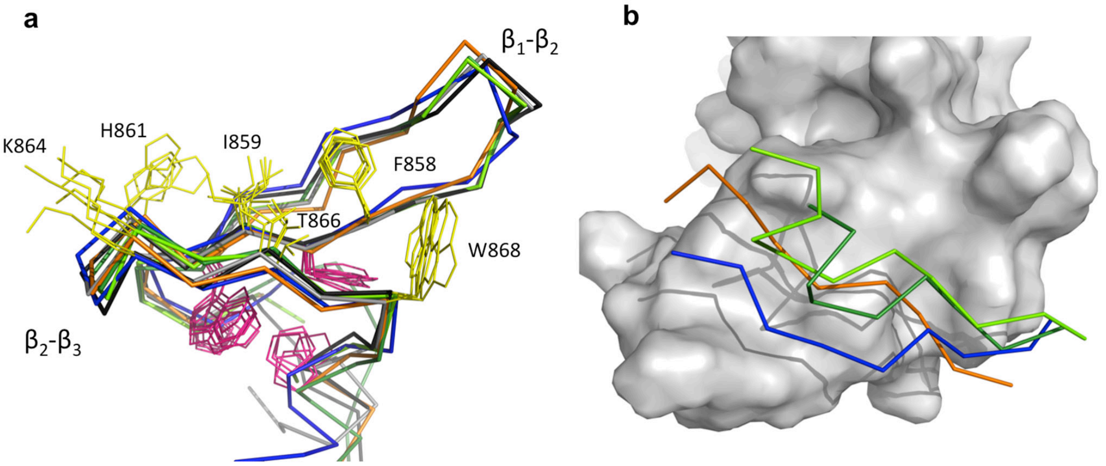


**Supplementary Figure S3. Structural comparison of different hNEDD4-WW3 complexes. (a)** Structural superposition of the hNEDD4-WW3 complexes with the HTLV1 (orange, 2KPZ) and Ebola-2KQ0 (blue, 2KQ0) Late domains, the ARRDC3 (dark green, 4N7H) and ENaC (light green, 2M30) cellular ligands and the NMR (grey) and Xray (black) structures of the apo domain (4N7F and 1WR7 respectively). Residues implicated in the hydrophobic buckle essential for domain stability as shown as magenta lines and residues conforming the xP and xY pockets as yellow lines. B) Variability in the orientation of the HTLV1 (orange), Ebola (blue) ARRDC3 (dark green) and ENaC (light green) ligands on the surface of the hNEDD4-WW3 domain.

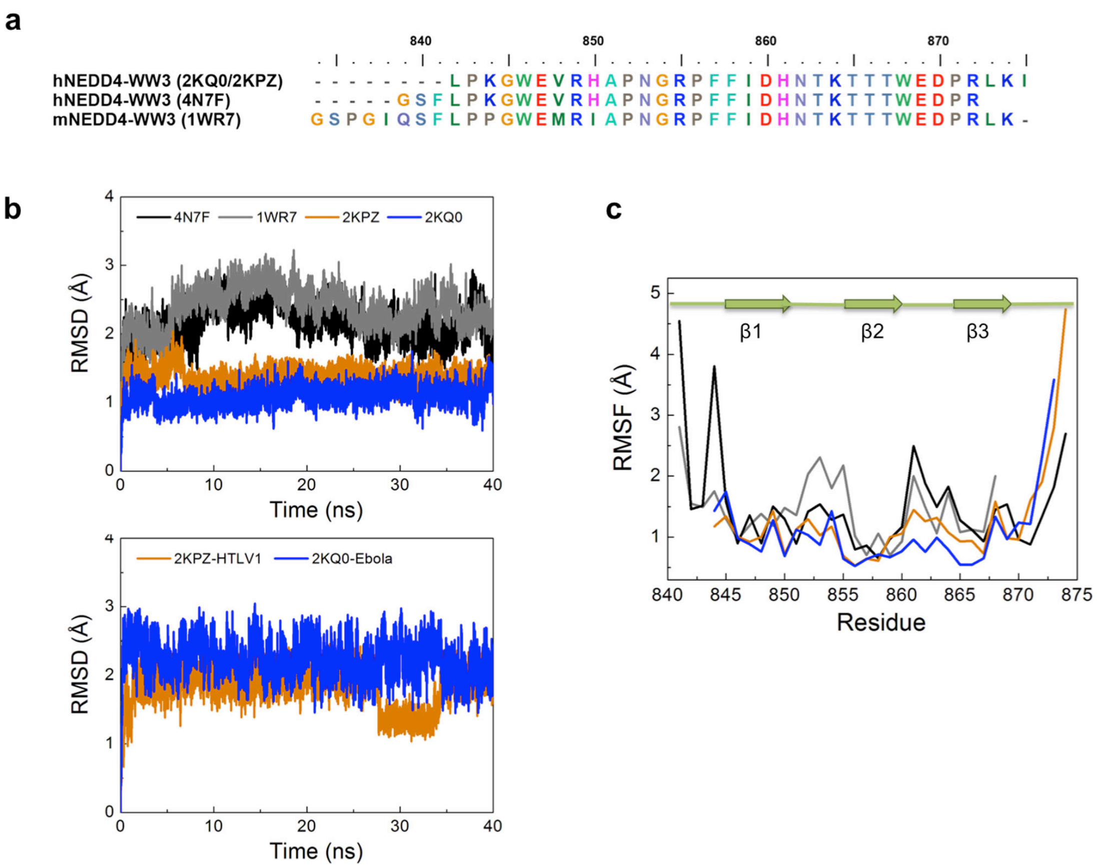


**Supplementary Figure S4. Molecular dynamics simulations of NEDD4-WW3. (a)** Sequence alignment of the NEDD4-WW3 constructs used in the MD simulations. **(b)** Time course of the backbone root mean square deviation (RMSD) for the free NEDD4-WW3 domain (4N7F: black lines and 1WR7: grey lines) and the Ebola (1KQ0: blue lines) and HTLV1 (1KPZ: orange lines) complexes. The RMSD values for the domain (upper panel) and the ligands (lower panel) are shown separately. To avoid distortions due to the highly unstructured N- and C- termini, RMSD calculations include residues 845-871 in the domain **C)** Root mean square fluctuations (RMSF) per residue for the apo-NEDD4-WW3 domain (4N7F as grey lines and 1WR7 as black lines), the HTLV1/NEDD4-WW3 complex (orange lines) and Ebola/NEDD4-WW3 complex (blue lines).

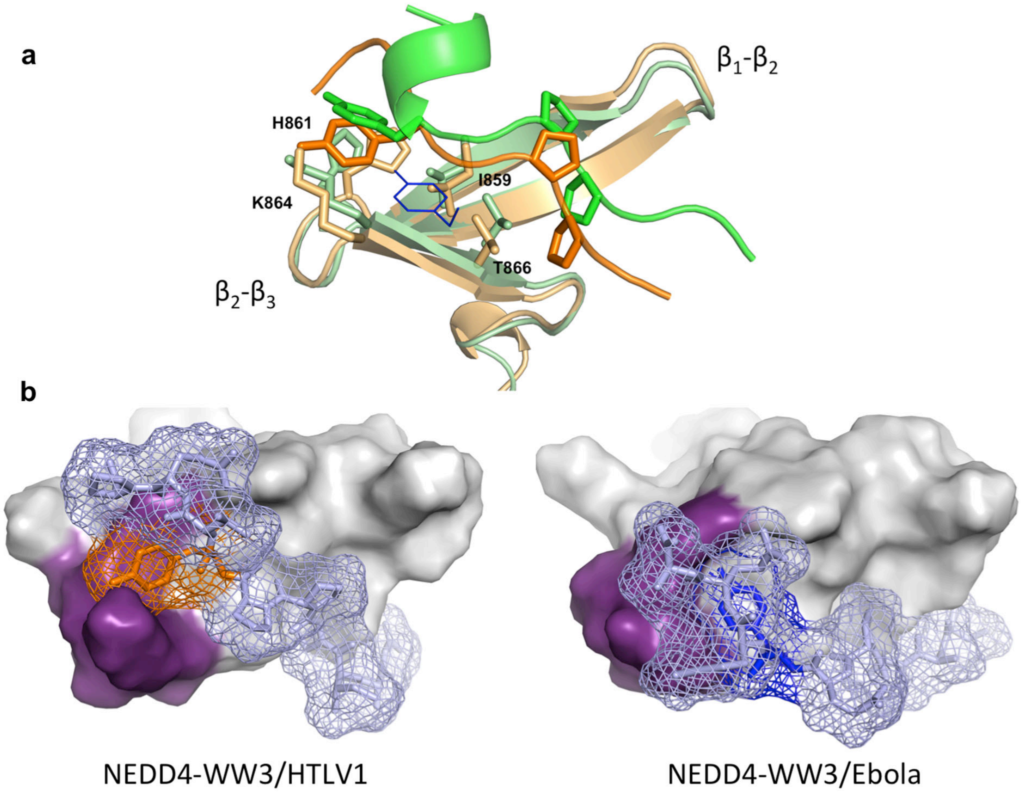


**Supplementary Figure S5. Ligand disposition at the binding site. (a)** Structural superposition of the HTLV1 (2KPZ, orange) and ENaC (2M30, green) complex structures showing a very similar configuration at the binding site. Shown are the cartoon representations of the NEDD4-WW3 domains, a ribbon representation of the two ligands and the stick representation of the most representative side chains in both the ligands ($P_{-2}$, $P_{-3}$ and $Y_0$) and the WW domains. The $Y_0$ residue from the Ebola complex is shown for comparison purposes as dark blue lines. **(b)** Packing of the HTLV1 (left) and Ebola (right) ligands at the binding site. Shown are surface representations of the WW domain and a mesh depiction the ligands highlighting the corresponding $Y_0$ side chains as orange (HTLV1) and blue (Ebola) sticks. Residues conforming the xY pocket are shown in purple.

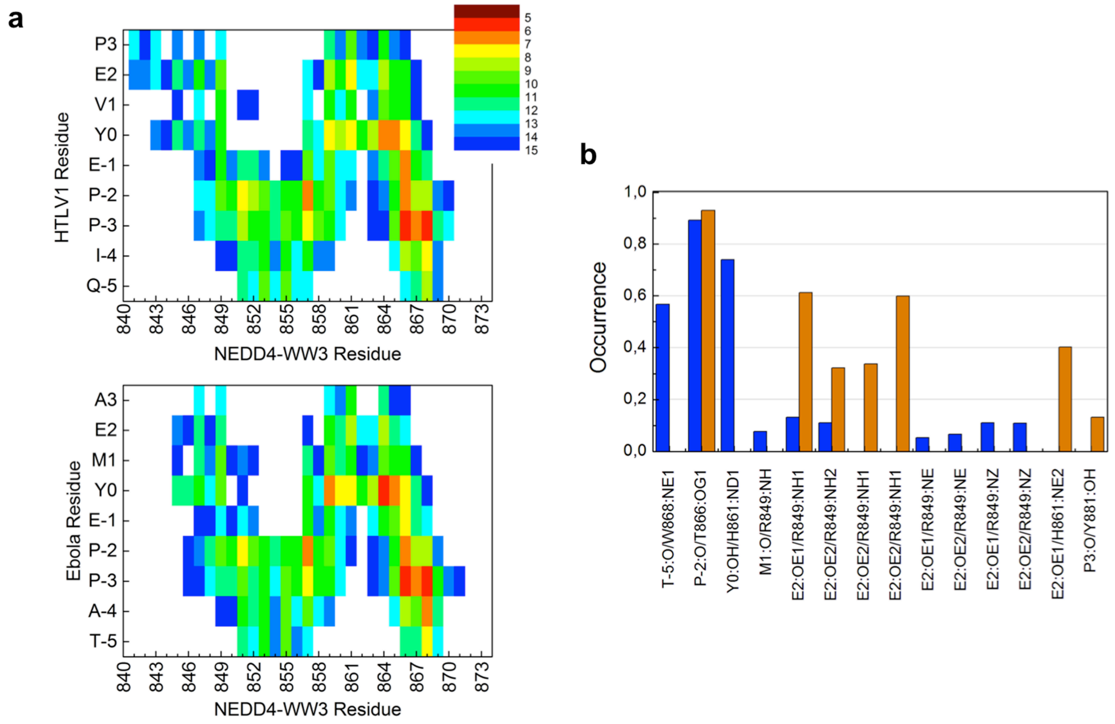


**Supplementary Figure S6.- Interfacial interactions in the MD trajectories of the Ebola and HTLV1 complexes with hNEDD4-WW3.** **(a)** Average contact distance between ligand residues and the hNEDD4-WW3 domain. Shown are the calculated average distances between the centroids of the ligand and domain residues colored according to a rainbow scheme. **(b)** Frequencies of occurrence of ligand-domain hydrogen bonds for the Ebola (blue) and HTLV1 (orange) complexes. Shown are all hydrogen bonds observed for more than 5% of the simulation time.

**Supplementary Table S1.** Sequences of Late domain peptides

| Name | Protein source | Sequence* |
|---|---|---|
| **HTLV1** | *Residues 13 to 24 from HTLV-1-Gag: Uniprot P14078* | $_{\mathbf{-8'}}\text{SDPQI}\mathbf{P}_{\mathbf{-3'}}\mathbf{P}_{\mathbf{-2'}}\mathbf{P}_{\mathbf{-1'}}\mathbf{Y}_{\mathbf{0'}}\text{VEP}_{3'}$ |
| **HTLV1-ter** | *Residues 18 to 27 from HTLV-1-Gag: Uniprot P14078* | $\mathbf{P}_{\mathbf{-3'}}\mathbf{P}_{\mathbf{-2'}}\mathbf{P}_{\mathbf{-1'}}\mathbf{Y}_{\mathbf{0'}}\text{VE}\underline{\text{PTAP}}_{6'}$ |
| **Ebola** | *Residues 5 to 16 from Zaire Ebola-VP40: Uniprot Q05128* | $_{\mathbf{-8'}}\text{IL}\underline{\text{PTA}\mathbf{P}_{\mathbf{-3'}}}\mathbf{P}_{\mathbf{-2'}}\mathbf{E}_{\mathbf{-1'}}\mathbf{Y}_{\mathbf{0'}}\text{MEA}_{\mathbf{3'}}$ |
| **Ebola-ter** | *Residues 5 to 13 from Zaire Ebola-VP40: Uniprot Q05128* | $_{\mathbf{-8'}}\text{IL}\underline{\text{PTA}\mathbf{P}_{\mathbf{-3}}}\mathbf{P}_{\mathbf{-2'}}\mathbf{E}_{\mathbf{-1'}}\mathbf{Y}_{\mathbf{0'}}$ |
| **Marburg** | *Residues 11 to 22 from Marburg-VP40: Uniprot P35260* | $_{\mathbf{-8'}}\text{MQYLN}\mathbf{P}_{\mathbf{-3'}}\mathbf{P}_{\mathbf{-2'}}\mathbf{P}_{\mathbf{-1'}}\mathbf{Y}_{\mathbf{0'}}\text{ADH}_{\mathbf{3'}}$ |
| **Rabies** | *Residues 30 to 41 from Rabies-M2: Uniprot P16287* | $_{\mathbf{-8'}}\text{DLWLP}\mathbf{P}_{\mathbf{-3'}}\mathbf{P}_{\mathbf{-2'}}\mathbf{E}_{\mathbf{-1'}}\mathbf{Y}_{\mathbf{0'}}\text{VPL}_{\mathbf{3'}}$ |
| **p53bp2** | *Residues 65 to 77 from Human p53 binding protein 2: Uniprot 13625* | $_{\mathbf{-9'}}\text{EYPPYP}\mathbf{P}_{\mathbf{-3'}}\mathbf{P}_{\mathbf{-2'}}\mathbf{P}_{\mathbf{-1'}}\mathbf{Y}_{\mathbf{0'}}\text{PSG}_{3'}$ |
| **PPPY** | *Binding consensus motif for class 1 WW domains [1]* | $\mathbf{P}_{\mathbf{-3'}}\mathbf{P}_{\mathbf{-2'}}\mathbf{P}_{\mathbf{-1'}}\mathbf{Y}_{\mathbf{0'}}$ |

* The PPxY core motif in the ligand sequence is marked in bold letters. A second PTAP Late motif, present in some ligands, has been underlined. Residues have been numbered according to the general recommendations for peptide ligands of modular domains [2] .

**Supplementary Table S2**. Comparison of the most relevant intermolecular NOEs between NEDD4-WW3 and the Ebola and HTLV1 Late domain ligands

| NEDD4-WW3 Residues | Protón | Residues | HTLV1 Proton | Ebola Proton |
|---|---|---|---|---|
| W868 | $H\varepsilon_1$ | Q-5´ ó T-5´ | $H\beta_1$, $H\beta_2$ | # $H\gamma$ |
| | $H\varepsilon_1$ | I-4´ ó A-4´ | $H\alpha$, $H\beta$, # $H\gamma$ | $H\alpha$, # $H\beta$ |
| | $H\delta_1$ | | $H\alpha$ | -- |
| | $H\zeta_2$ | | -- | $H\alpha$, # $H\beta$ |
| | $H\varepsilon_1$ | **P-3´** | $H\delta_1$, $H\delta_2$ | $H\alpha$, $H\delta_1$, $H\delta_2$ |
| | $H\delta_1$ | | $H\delta_1$, $H\delta_2$ | $H\delta_1$, $H\delta_2$ |
| | $H\zeta_2$ | | $H\alpha$, $H\delta_1$, $H\delta_2$ | $H\alpha$, $H\delta_1$, $H\delta_2$ |
| F857 | #$H\delta$ | **P-2´** | $H\delta_1$, $H\delta_2$, $H\beta_1$, | $H\delta_1$, $H\delta_2$, $H\beta_1$, |
| | #$H\varepsilon$ | | $H\beta_1$, $H\beta_2$, $H\gamma_1$, $H\gamma_2$ | $H\beta_1$, $H\beta_2$, $H\gamma_1$, $H\gamma_2$ |
| I859 | #$H\gamma_1$ | **Y0´** | #$H\delta$ | #$H\varepsilon$ |
| | #$H\gamma_2$ | | #$H\delta$, #$H\varepsilon$ | #$H\delta$, #$H\varepsilon$ |
| H861 | HN | | -- | #$H\varepsilon$ |
| | $H\alpha$ | | #$H\varepsilon$ | #$H\varepsilon$ |
| | #$H\varepsilon$ | | #$H\varepsilon$ | #$H\varepsilon$ |
| K864 | $H\alpha$ | | #$H\varepsilon$ | #$H\varepsilon$, # $H\delta$ |
| | $H\beta_1$ | | #$H\varepsilon$, #$H\delta$ | #$H\varepsilon$, #$H\delta$ |
| | $H\beta_2$ | | #$H\varepsilon$, #$H\delta$ | #$H\varepsilon$, #$H\delta$ |
| | $H\delta_1$ | | #$H\varepsilon$ | #$H\varepsilon$ |
| | $H\delta_2$ | | #$H\varepsilon$ | #$H\varepsilon$ |
| | $H\gamma_1$ | | #$H\varepsilon$ | #$H\varepsilon$ |
| | $H\gamma_2$ | | #$H\varepsilon$ | #$H\varepsilon$ |
| T865 | HN | | -- | $H\beta_1$, $H\beta_2$ |
| | $H\alpha$ | | -- | $H\beta_1$, $H\beta_2$ |
| T866 | #$H\gamma_2$ | | HN, $H\alpha$, $H\beta_1$, | HN, $H\alpha$, $H\beta_1$, |
| R849 | $H\varepsilon$ | E2´ | $H\gamma_2$, $H\gamma_3$ | $H\beta_2$, $H\gamma_2$ |

# Protons considered chemically equivalent.

**Supplementary Table S3**. YASARA[3] analysis of intermolecular interactions in NEDD4-WW3 complexes with Ebola and HTLV1 Late domain peptides.

| **Hydrogen bonds** | | | | | |
|---|---|---|---|---|---|
| **Ebola/NEDD4-WW3** | | | **HTLV1/NEDD4-WW3** | | |
| **Atoms** | **Distance (Å)** | **Energy (kJ·mol$^{-1}$)** | **Atoms** | **Distance (Å)** | **Energy (kJ·mol$^{-1}$)** |
| | | | Q-5´:O-W868:NE1 | 2.04 | 25.00 |
| | | | P-3´:O-T866:OG1 | 1.80 | 21.50 |
| | | | Y0:O-K864:NZ | 1.77 | 21.00 |
| E2´:OE1-R849:NH2 | 1.68 | 21.88 | E2´:OE1-R849:NH2 | 1.64 | 25.00 |
| E2´:O-H861:ND1 | 2.07 | 17.00 | P3´:OT2-H861:ND1 | 1.70 | 17.78 |
| **Hydrophobic interactions** | | | | | |
| **Ebola/NEDD4-WW3** | | | **HTLV1/NEDD4-WW3** | | |
| **Residues** | **# Interact** | **Strength** | **Residues** | **# Interact** | **Strength** |
| | | | Q-5´/W868 | 1 | 0.276 |
| P-3´/F857 | 2 | 1.840 | P-3´/F857 | 2 | 1,079 |
| P-3´/ W868 | 2 | 1.169 | P-3´/ W868 | 4 | 3,271 |
| P-3´/ T866 | 1 | 0.975 | P-1´/ T866 | 1 | 0.991 |
| Y0´/ K864 | 2 | 1.336 | Y0´/ I859 | 2 | 1,545 |
| Y0´/ T866 | 1 | 0.951 | Y0´/ I861 | 2 | 1,966 |
| **Cation-π interactions** | | | | | |
| **Ebola/NEDD4-WW3** | | | **HTLV1/NEDD4-WW3** | | |
| **Residues** | **# Interact** | **Strength** | **Residues** | **# Interact** | **Strength** |
| | | | Y0´/ K864 | 1 | 1.000 |

**Supplementary Table S4**. $\chi$1 dihedral angles for Thr 866 and Ile/Val 859 in Class I WW domain complexes

| **WW domains from E3 ubiquitin ligases** | | | | | | |
|---|---|---|---|---|---|---|
| **PDB-ID** | **Complex** | **Ligand** | **Kd** | **T866-$\chi$1** | **I859-$\chi$1** | **Type** |
| 4N7F[4] | NEDD4-WW3 (human) | -- | | -54.5/-54.3 | -60.1/60.1 | -- |
| 2KPZ | HTLV1/hNEDD4-WW3 | QIPPPYVEP | 61 µM | +41,1 | -45.7 | I-HTLV |
| 2KQ0 | Ebola/hNEDD4-WW3 | TAPPEYMEA | 147 µM | -176,5 | +42.9 | II-Ebola |
| 2M30[6] | αENaC/hNEDD4-WW3 | TAPPPAYATLG | 45 µM | +39,2 | -44.7 | I-HTLV |
| 4N7H[4] | ARRDC3h/hNEDD4-WW3 | EAPPSYAEV | 3,3 µM | +67.7 | -60.3 | I-HTLV |
| 2EZ5[7] | Comm/dNEDD4-WW3 | TGLPSYDEALH | 3,1 µM | +65,1 | -64.8 | I-HTLV |
| 1I5H[5] | βENaC/hNEDD4-WW3 | GSTLPIPGTPPPNYDSL | 21 µM | +173.1 | +178.3 | III |
| 2N8T [8] | CX43-Cter/rNEDD4-WW2 | APLpSPMpSPPGYKLV | 430 µM | -79,2 | +55,4 (V) | III |
| 2MPT [9] | HECT-Cterm/hNEDD4L-WW3 | RLDLPPYETFEDI | 4,0 µM | +45,0 | -72,2 | II-Ebola |
| 2LTY[10] | Smad7/NEDD4L-WW2 | ELESPPPPYSRYPMD | 4,2 µM | +175.8 | -66.8 (V) | II-Ebola |
| 2JO9 [11] | Epstein-Barr/hITCHY-WW3 | EEPPPPYED | 52 µM | +40.8 | +24.4 (V) | II-Ebola |
| 5DWS | TXNYP/hITCHY-WW3 | PEAPPCYMDVI | ----- | +64,5 | -174,7 (V) | III |
| 5DZD | TXNYP/hITCHY-WW4 | TPEAPPCYMDVI | ----- | +58,8 | 175,8 (V) | III |
| 2JMF[12] | Notch/SU(dx)-WW4 | NTGAKQPPSYEDCIK | 45 µM | +50,7 | +176,9 | I-HTLV |
| 4LCD[13] | Sna3/RSP5-WW3 | AQPPAYDEDDE | ----- | +56,5 | -173,7 | III |
| 2LB1[14] | Smad1/SMURF1-WW2 | DTPPPAYLPPEDP | ----- | +52.8 | -69.6 (V) | I-HTLV |
| 2LTX[10] | Smad7/SMURF1-WW2 | ELESPPPPYSRYPMD | 4,2 µM | -51.1 | -68.7 (V) | III |
| 2LTZ[10] | Smad7/SMURF2-WW3 | ELESPPPPYSRYPMD | 4,5 µM | -171.7 | -76.7 (V) | III |
| 2DJY[15] | Smad7/SMURF2-WW3 | GPLGSELESPPPPYSRY | 40 µM | +42.9 | -166,6 (V) | III |
| **YAP WW domains** | | | | | | |
| **PDB-ID** | **Complex** | **Ligand** | | **T866-$\chi$1** | **I859-$\chi$1** | **Type** |
| 1K9R [16] | PLPPY/hYAP-WW1 | PLPPY | 500 µM | +64,6 | -153,9 | II-Ebola |
| 1K9Q [16] | GPPPY/hYAP-WW1 | GPPPY | 700 µM | +67,8 | -85,6 | I-HTLV |
| 1JMQ [16] | WBP1/hYAP-WW1 | GTPPPPYTVG | 40 µM | +54,8 | -68,4 | III |
| 2LTV [10] | Smad7/hYAP-WW2 | ELESPPPPYSRYPMD | 60 µM | -30,3 | 47,2 | II-Ebola |
| 2LTW [10] | Smad7/hYAP-WW1 | ELESPPPPYSRYPMD | 6,9 µM | -42,8 | -90,3 | III |
| 2LAW[14] | Smad1/hYAP-WW2 | DTPPPAYLPPEDP | ----- | -57,0 | 60,3 | III |

**Supplementary Table S5.-** Structure-based prediction of binding enthalpies for WW domain complexes

| ***Ligand*** | ***$\Delta H_{exp}$ (25C) (kJ·mol$^{-1}$)*** | ***$\Delta ASA_{ap}$ (Å$^2$)*** | ***$\Delta ASA_{pol}$ (Å$^2$)*** | ***$\Delta H_{int}$ (25C) (kJ·mol$^{-1}$)*** | ***$\Delta H_{conf}$ (kJ·mol$^{-1}$)*** |
|---|---|---|---|---|---|
| **Ebola (2KQ0)** | -50,7 | -492,85 | -275,98 | -20,69 | -30,01 |
| **HTLV1 (2KPZ)** | -68,2 | -549,66 | -270,51 | -18,23 | -49,97 |
| **ARRDC3 (4N7H)** | -75,4 | -571,42 | -217,86 | -10,73 | -64,67 |
| **ENaC (2M30)** | -58,2 | -536,29 | -203,23 | -9,91 | -48,29 |

*Binding enthalpies were calculated from the corresponding high resolution structures according to the equation: $\Delta H_{binding}$ (25 ºC) = $\Delta H_{conf}$ (25 ºC) + $\Delta H_{int}$ (25 ºC) = $\Delta H_{conf}$ (25 ºC) – 7,35· $\Delta ASA_{ap}$ + 31,06· $\Delta ASA_{pol}$, as described in* [17]

## REFERENCES


1 Otte, L. *et al.* WW domain sequence activity relationships identified using ligand recognition propensities of 42 WW domains. *Protein science : a publication of the Protein Society* **12**, 491-500, doi:10.1110/ps.0233203 (2003).

2 Aasland, R. *et al.* Normalization of nomenclature for peptide motifs as ligands of modular protein domains. *FEBS letters* **513**, 141-144 (2002).

3 Krieger, E. & Vriend, G. YASARA View - molecular graphics for all devices - from smartphones to workstations. *Bioinformatics* **30**, 2981-2982, doi:10.1093/bioinformatics/btu426 (2014).

4 Qi, S., O'Hayre, M., Gutkind, J. S. & Hurley, J. H. Structural and biochemical basis for ubiquitin ligase recruitment by arrestin-related domain-containing protein-3 (ARRDC3). *J Biol Chem* **289**, 4743-4752, doi:M113.527473 [pii] 10.1074/jbc.M113.527473 (2014).

5 Kanelis, V., Rotin, D. & Forman-Kay, J. D. Solution structure of a Nedd4 WW domain-ENaC peptide complex. *Nat Struct Biol* **8**, 407-412, doi:10.1038/87562 87562 [pii] (2001).

6 Bobby, R. *et al.* Structure and dynamics of human Nedd4-1 WW3 in complex with the alphaENaC PY motif. *Biochim Biophys Acta* **1834**, 1632-1641, doi:S1570-9639(13)00185-4 [pii] 10.1016/j.bbapap.2013.04.031 (2013).

7 Kanelis, V., Bruce, M. C., Skrynnikov, N. R., Rotin, D. & Forman-Kay, J. D. Structural determinants for high-affinity binding in a Nedd4 WW3* domain-Comm PY motif complex. *Structure* **14**, 543-553, doi:10.1016/j.str.2005.11.018 (2006).

8 Spagnol, G. *et al.* Structural Studies of the Nedd4 WW Domains and Their Selectivity for the Connexin43 (Cx43) Carboxyl Terminus. *J Biol Chem* **291**, 7637-7650, doi:10.1074/jbc.M115.701417 (2016).

9 Escobedo, A. *et al.* Structural basis of the activation and degradation mechanisms of the E3 ubiquitin ligase Nedd4L. *Structure* **22**, 1446-1457, doi:10.1016/j.str.2014.08.016 (2014).

10 Aragon, E. *et al.* Structural Basis for the Versatile Interactions of Smad7 with Regulator WW Domains in TGF-beta Pathways. *Structure* **20**, 1726-1736, doi:S0969-2126(12)00279-1 [pii] 10.1016/j.str.2012.07.014 (2012).

11 Morales, B. *et al.* NMR structural studies of the ItchWW3 domain reveal that phosphorylation at T30 inhibits the interaction with PPxY-containing ligands. *Structure* **15**, 473-483, doi:S0969-2126(07)00112-8 [pii] 10.1016/j.str.2007.03.005 (2007).

12 Jennings, M. D., Blankley, R. T., Baron, M., Golovanov, A. P. & Avis, J. M. Specificity and autoregulation of Notch binding by tandem WW domains in suppressor of Deltex. *J Biol Chem* **282**, 29032-29042, doi:10.1074/jbc.M703453200 (2007).

13 Kamadurai, H. B. *et al.* Mechanism of ubiquitin ligation and lysine prioritization by a HECT E3. *eLife* **2**, e00828, doi:10.7554/eLife.00828 (2013).

14 Aragon, E. *et al.* A Smad action turnover switch operated by WW domain readers of a phosphoserine code. *Genes Dev* **25**, 1275-1288, doi:25/12/1275 [pii] 10.1101/gad.2060811 (2011).

15 Chong, P. A., Lin, H., Wrana, J. L. & Forman-Kay, J. D. An expanded WW domain recognition motif revealed by the interaction between Smad7 and the E3 ubiquitin ligase Smurf2. *J Biol Chem* **281**, 17069-17075, doi:M601493200 [pii] 10.1074/jbc.M601493200 (2006).
16 Pires, J. R. *et al*. Solution structures of the YAP65 WW domain and the variant L30 K in complex with the peptides GTPPPPYTVG, N-(n-octyl)-GPPPY and PLPPY and the application of peptide libraries reveal a minimal binding epitope. *J Mol Biol* **314**, 1147-1156, doi:10.1006/jmbi.2000.5199 S0022-2836(00)95199-4 [pii] (2001).
17 Luque, I. & Freire, E. Structural parameterization of the binding enthalpy of small ligands. *Proteins* **49**, 181-190, doi:10.1002/prot.10208 (2002).
